# Structural and Functional Recovery of Sensory Cilia in *C*. *elegans* IFT Mutants upon Aging

**DOI:** 10.1371/journal.pgen.1006325

**Published:** 2016-12-01

**Authors:** Astrid Cornils, Ashish K. Maurya, Lauren Tereshko, Julie Kennedy, Andrea G. Brear, Veena Prahlad, Oliver E. Blacque, Piali Sengupta

**Affiliations:** 1 Department of Biology and National Center for Behavioral Genomics, Brandeis University, Waltham, Massachusetts; 2 School of Biomolecular and Biomedical Science, University College Dublin, Belfield, Dublin, Ireland; 3 Department of Biology, Aging Mind and Brain Initiative, University of Iowa, Iowa City, Iowa; Washington University School of Medicine, UNITED STATES

## Abstract

The majority of cilia are formed and maintained by the highly conserved process of intraflagellar transport (IFT). Mutations in IFT genes lead to ciliary structural defects and systemic disorders termed ciliopathies. Here we show that the severely truncated sensory cilia of hypomorphic IFT mutants in *C*. *elegans* transiently elongate during a discrete period of adult aging leading to markedly improved sensory behaviors. Age-dependent restoration of cilia morphology occurs in structurally diverse cilia types and requires IFT. We demonstrate that while DAF-16/FOXO is dispensable, the age-dependent suppression of cilia phenotypes in IFT mutants requires cell-autonomous functions of the HSF1 heat shock factor and the Hsp90 chaperone. Our results describe an unexpected role of early aging and protein quality control mechanisms in suppressing ciliary phenotypes of IFT mutants, and suggest possible strategies for targeting subsets of ciliopathies.

## Author Summary

Cilia are ‘antenna-like’ structures that are present on nearly all cell types in animals. These structures are important for sensing and signaling external cues to the cell. Most cilia are formed by a protein transport process called ‘intraflagellar transport’ or IFT. Mutations in IFT genes result in severe cilia defects, and are causal to a large number of diverse human disorders called ciliopathies. Since the genes and processes by which cilia are formed are similar across species, studies in experimental models such as the nematode *C*. *elegans* can greatly inform our overall understanding of cilia formation and function. Here we report the surprising observation that the structures and functions of severely defective cilia in nematodes with disrupted IFT genes markedly improve upon aging. We find that protein quality control mechanisms that normally decline in aging are required for this age-dependent recovery of cilia structure. Our results raise the possibility that the effects of some mutations in IFT genes can be bypassed under specific conditions, thereby restoring cilia functions.

## Introduction

**Fig 1:**
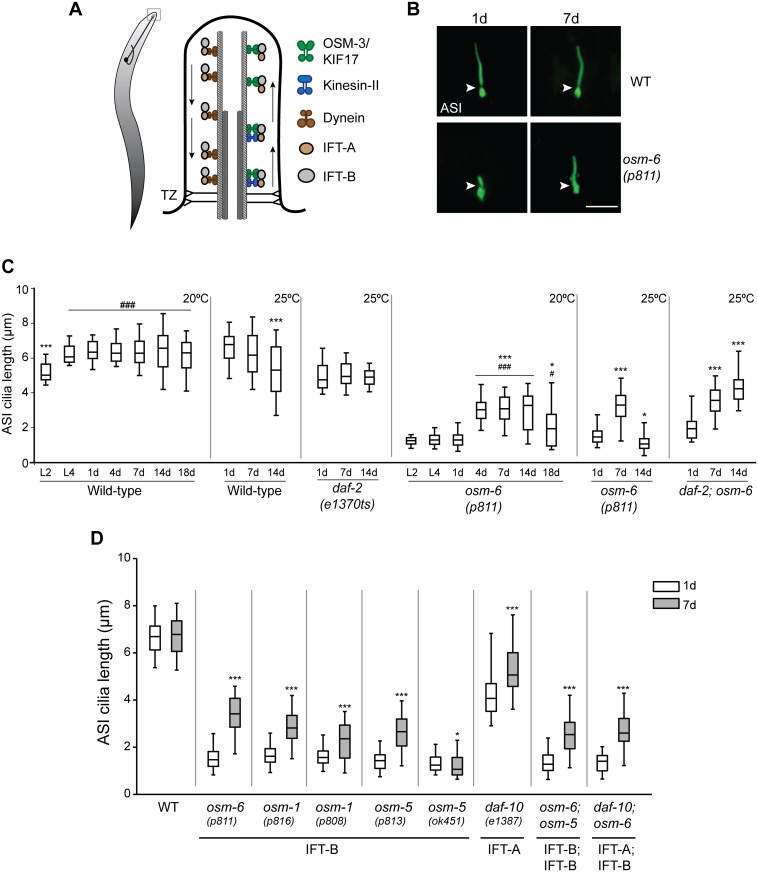
Cilia of the ASI sensory neurons elongate in aged IFT mutants. **(A)** (Left) Cartoon of a worm showing a representative sensory neuron in the worm head. Cilia are present at the dendritic ends at the nose (box). (Right) Diagrammatic representation of the structure of a typical cilium and IFT in *C*. *elegans*. Arrows indicated direction of IFT. TZ—transition zone (showing Y-link microtubule-to-membrane connectors). **(B)** Representative images of ASI cilia in 1d and 7d old adult wild-type (WT) and *osm-6(p811)* mutants. Arrowheads indicate the cilia base. Anterior is at top. ASI cilia were visualized via expression of a GFP-tagged SRG-36 GPCR protein expressed under the ASI-specific *str-3* promoter. Scale bar: 5 μm. **(C)** Quantification of ASI cilia length in animals of the indicated genetic backgrounds at different larval stages (L2, L4) or days of adulthood. Horizontal lines indicate 25^th^, 50^th^ and 75^th^ percentiles; bars indicate 5^th^ and 95^th^ percentiles. * and *** indicate different from 1d within a genotype at *P*<0.05 and 0.001, respectively; ^#^ and ^###^ indicate different from L2 within a genotype at *P*<0.05 and 0.001, respectively (Kruskal-Wallis test with post hoc paired comparisons). n>30 for each; ≥3 independent experiments. Animals were grown at either 20°C or 25°C for each set of experiments (indicated at top right). **(D)** Quantification of ASI cilia length in 1d and 7d old animals of the indicated genotypes grown at 20°C. ASI cilia were visualized via expression of *str-3*p::*srg-36*::*gfp*. Alleles used in the double mutant strains were *osm-6(p811)*, *osm-5(p813)*, and *daf-10(e1387)*. Horizontal lines indicate 25^th^, 50^th^ and 75^th^ percentiles; bars indicate 5^th^ and 95^th^ percentiles. * and *** indicate different from 1d within a genotype at *P*<0.05 and 0.001, respectively (Wilcoxon Mann-Whitney U test). n>30 for each; ≥3 independent experiments.

The coordinated functions of multiple proteins in large macromolecular complexes is essential for many fundamental cellular processes including the building of multicomponent cellular structures. For instance, primary cilia, which are microtubule-based sensory organelles present on nearly all metazoan cells, are generated and maintained by large protein complexes that mediate the conserved process of intraflagellar transport (IFT). These IFT complexes link cargo molecules to kinesin-2 and cytoplasmic dynein 1b molecular motors to build these critical signaling structures [1–3] (Fig 1A). While null mutations in IFT genes result in severe disruption or loss of cilia, and embryonic lethality in vertebrates [4–6], hypomorphic mutations in core IFT genes lead to weaker cilia structural defects and tissue-specific phenotypes, characteristic of syndromes collectively termed ciliopathies [7–12]. Thus, identifying conditions that suppress and/or bypass deleterious effects of IFT gene mutations and restore cilia growth is of great interest and medical relevance.

Cilia are present at the dendritic endings of a subset of sensory neurons in *C*. *elegans* (Fig 1A) [13, 14]. As in other animals, IFT is essential for ciliogenesis in *C*. *elegans*, and IFT-A and IFT-B core complex proteins are highly conserved [15]. Mutations in IFT-A complex genes such as *daf-10/*IFT122 result in accumulation of proteins at the cilia tips suggesting defects in retrograde transport, whereas mutations in core IFT-B genes such as *osm-6/*IFT52 and *osm-5*/IFT88 affect anterograde transport leading to severely truncated cilia with protein accumulation at the ciliary base [14, 16]. In both cases, sensory neuronal function is severely impaired [17–19]. Thus, *C*. *elegans* provides an excellent experimental system in which to identify and analyze mechanisms of ciliogenesis and cilia function.

*C*. *elegans* is also an established model organism for the study of aging [20–22]. Work in multiple systems has demonstrated that aging is a highly regulated process that is under tight genetic control [23–25]. A hallmark of aging is the decreased ability to maintain protein function or protein homeostasis (proteostasis), which results in increased cellular damage and decline of cellular and organismal functions [26–29]. Compromised proteostasis in aged animals is in part due to reduced functionality of protein quality control mechanisms, thereby enhancing aggregation and accumulation of misfolded proteins [30–34]. Thus, protein complexes such as IFT particles that rely on defined stoichiometry of individual components [3, 35, 36] may be particularly vulnerable to aging. However, how aging affects primary cilia structure and function has not been examined in detail.

Here we show that aging leads to transient structural and functional recovery of severely defective sensory cilia in hypomorphic IFT mutants in *C*. *elegans*. This age-dependent improvement of cilia morphology and properties occurs in multiple unique cilia types and is IFT-dependent. We find that the HSF1 heat shock factor, the Hsp90 molecular chaperone, and the ubiquitin-proteasome system are required for the observed suppression of cilia structural and functional defects in IFT mutants. Our results describe a protective role of early aging and protein quality control mechanisms in restoring sensory cilia function in hypomorphic IFT mutants, and raise the possibility that related mechanisms may similarly ameliorate cilia defects and improve cellular and organismal homeostasis in other contexts.

## Results

### Cilia morphology is altered in late stages of aging

To investigate whether aging perturbs cilia structure and function in *C*. *elegans*, we began by examining the simple rod-like cilia of the ASI sensory neuron pair in the head amphid organs [13, 14, 37]. ASI cilia were visualized via cell-specific expression of a GFP-tagged SRG-36 pheromone receptor protein, which localizes specifically to ciliary membranes [38] (Fig 1B). We found that ASI cilia reached their final length by the L4 larval stage, suggesting that cilium length is not grossly affected by animal size following transition into adulthood (Fig 1C). However, we noted increased variability in ASI cilia length as animals aged (Fig 1C), suggesting that cilium length may be affected upon aging.

To further investigate this issue, we compared ASI cilia length in wild-type and the long-lived *daf-2(e1370ts)* [39, 40] insulin receptor mutants. Insulin/IGF-1 signaling is the major pathway that regulates aging in *C*. *elegans* as well as in other species [41, 42]. At the restrictive growth temperature of 25°C, wild-type and *daf-2(e1370ts)* mutants exhibit a mean lifespan of ~15d and ~23d, respectively [41]. ASI cilia length was more variable and on average, significantly shorter in 14d old compared to 1d old wild-type animals at this temperature (Fig 1C). In contrast, although ASI cilia were shorter in *daf-2(e1370ts)* mutants for unknown reasons, their length remained constant through 14d of adulthood at this temperature (Fig 1C). We conclude that ASI cilia length becomes more variable in old age, and that this phenotype is under genetic regulation.

### Aging partly restores cilia morphology in hypomorphic IFT mutants

Loss of function mutations in IFT-B complex genes such as *osm-6*/IFT52 result in severely truncated cilia [14, 43] (Fig 1B and 1C). Unexpectedly, we observed that the severely truncated ASI cilia in *osm-6(p811)* mutants lengthened in adults during early aging (Fig 1B and 1C). While these cilia did not elongate to wild-type lengths, they were nevertheless nearly twice as long in 4-7d old, as compared to 1d old, *osm-6* adults grown at 20°C (Fig 1C). The cilia did not elongate with further aging and were shortened in older animals (Fig 1C). We observed similar elongation of ASI cilia in 7d old *osm-6* mutants grown at 25°C; these cilia were significantly shortened by 14d (Fig 1C). In contrast, the elongated cilia were maintained in 14d old *daf-2;osm-6* double mutants (Fig 1C), consistent with delayed aging in these animals. The observed age-dependent elongation was not specific to the *osm-6(p811)* allele, since similar effects were observed in the *osm-6(m533)* mutant (S1A Fig). We also observed ASI cilia elongation upon visualization of cilia using a soluble fluorescent reporter protein (S1B Fig), indicating that the quantified ASI cilia length was not an artifact due to altered SRG-36 receptor protein trafficking or ciliary localization at different ages. These results indicate that the effects of *osm-6* mutations on ASI cilia can be partially and transiently suppressed during early stages of aging.

OSM-6/IFT52 comprises a core component of the larger IFT-B protein complex [3, 16, 35, 43]. We asked whether cilia elongation with age is specific to mutations in *osm-6*, or is a general phenomenon observed in animals mutant for other IFT-B complex genes. Cilia elongation was observed to a similar extent in 7d old *osm-1/*IFT172 and *osm-5/*IFT88 IFT-B complex component mutants grown at 20°C (Fig 1D). Cilia in IFT-A complex genes are not shortened to the same extent as in IFT-B complex mutants [14]. Nevertheless, we also observed a lengthening of ASI cilia in 7d old *daf-10/*IFT122 IFT-A mutants (Fig 1D). Moreover, ASI cilia in *osm-6;osm-5* and *daf-10;osm-6* double mutants also elongated significantly with age (Fig 1D). Henceforth, we refer to this phenomenon as age-dependent cilia recovery (AdCR).

In the process of characterizing these IFT mutants, we noted that many of the isolated IFT mutant alleles in *C*. *elegans* are likely to be hypomorphs. These include the canonical *osm-6(p811)* allele which appears to encode a cilia-localized protein likely translated from a secondary or cryptic start codon (S2A–S2C Fig), the *osm-1(p816)* allele which is an in-frame deletion predicted to encode a protein lacking 2 of the 14 TPR repeats, and the *osm-5(p813)* allele which is a nonsense mutation encoding a truncated protein that contains the coiled-coil domain and 5 of the 12 TPR repeats (S2A Fig). We asked whether AdCR occurs only in strains carrying partial loss of function mutations in IFT genes, or whether the ciliary phenotypes of null mutations in these genes can also be bypassed upon aging. The *osm-5(ok451)* mutation is a large deletion/insertion in the gene, and is likely a null allele (S2A Fig). Interestingly, we noted that the ASI cilia failed to elongate in aged *osm-5(ok451)* mutants, and instead were significantly shorter (Fig 1D). These observations indicate that AdCR can occur in animals with compromised, but not entirely absent, IFT proteins.

### The cilia of multiple sensory neuron types exhibit AdCR in IFT mutants

**Fig 2:**
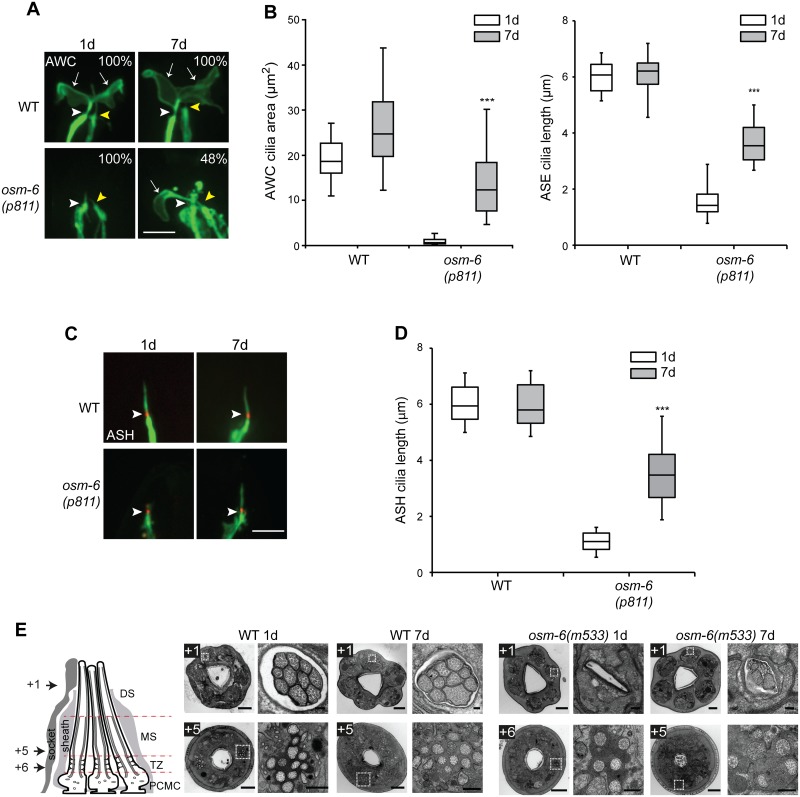
Cilia of multiple sensory neurons exhibit structural recovery in aged IFT mutants. **(A)** Representative images of the fan-shaped AWC, and rod-like ASE, cilia in 1d and 7d old wild-type and *osm-6(p811)* mutants. Cilia were visualized via expression of *ceh-36*p::*gfp* which drives expression in AWC and ASE [44]. White and yellow arrowheads mark the bases of the AWC and ASE cilia, respectively. Arrows mark the AWC cilia membraneous expansions (‘fans’). Numbers indicate the percentage of animals exhibiting phenotypes similar to those shown; n≥12 for each. Anterior is at top. Scale bar: 5 μm. **(B)** Quantification of AWC cilia fan area (left) and ASE cilia length (right) in 1d and 7d old wild-type and *osm-6(p811)* mutants. *** indicates different from 1d within a genotype at *P*<0.001 (Wilcoxon Mann-Whitney U test). n>30 each; 3 independent experiments. **(C)** Representative images of ASH cilia in 1d and 7d old adult wild-type (WT) and *osm-6(p811)* mutants. Arrowheads indicate the cilia base. ASH neuronal processes including cilia are marked via expression of GFP under the *sra-6* promoter. The cilium base is marked via localization of MKSR-2::TagRFP. Anterior is at top. Scale bar: 5 μm. **(D)** Quantification of ASH cilia length in wild-type and *osm-6(p811)* mutants at the indicated days of adulthood. *** indicates different from 1d within a genotype at *P*<0.001 (Wilcoxon Mann-Whitney U test). n>30 for each; ≥3 independent experiments. **(E)** Transmission electron microscopy of amphid channel sensory pores in adult 1d and 7d animals. Images acquired from serial cross sections of wild-type and *osm-6(m533)* mutant worms; each image pair consists of a low magnification image of the entire nose tip (left) and a high magnification image of an amphid pore (right; boxed regions in images at left). Numbers (microns) denote proximal positioning of section relative to the distal-most first section in the series; section positions also indicated in schematic. The schematic is a longitudinal representation of a wild-type amphid neuronal pore, enveloped by supporting sheath and socket glial cells. Pores consist of 10 ciliary axonemes (only 3 are shown), each with distal segment (DS; singlet microtubules), middle segment (MS; doublet microtubules), transition zone (TZ) and periciliary membrane (PCMC) subcompartments. Bars; 2 μm (low magnification images), 200 nm (high magnification images).

We next asked if AdCR is restricted to ASI cilia by examining the cilia of additional sensory neurons in IFT-B mutants. We visualized and quantified cilia morphologies in three other head amphid sensory neurons (AWC, ASE and ASH) via expression of neuron-specific cilia markers. While ASH and ASE cilia are simple and rod-like in shape similar to ASI cilia, AWC cilia exhibit complex membranous morphologies and are ensheathed by glial cells [14, 37]. We observed marked restoration of the morphologies of all three cilia types in 7d old *osm-6* adults (Fig 2A–2D), indicating that AdCR is a general feature in multiple ciliated neuron types.

To determine how closely these recovered cilia resemble their wild-type counterparts, we examined their ultrastructure via serial section electron microscopy. The cilia of eight pairs of amphid sensory neurons, including the ASI neurons, comprise ten axonemes that are bundled together and exposed to the environment via a channel created by the amphid socket and sheath glial cells (channel cilia) [14, 37]. Each cilium harbors a proximal region (transition zone and middle segments) containing 9 outer doublet microtubules and a distal region (distal segments) containing 9 outer singlet microtubules (Fig 2E, S3A and S3C Fig) [14, 37]. In IFT-B mutants such as *osm-6(p811)* that exhibit severely truncated cilia, the amphid channel is frequently deformed making it challenging to identify and visualize ciliary ultrastructure. We, therefore, examined *osm-6(m533)* mutants in which the amphid channel and cilia lengths are affected to a lesser degree (S1A Fig). Few if any axonemes were observed in distal parts of the channel in 1d old *osm-6(m533)* mutants (Fig 2E, S3A, S3C and S3D Fig), consistent with the shortened cilia in these animals (S1A Fig). However, axonemes were consistently observed in distal sections of 7d old *osm-6* animals (Fig 2E, S3A–S3D Fig), indicating that the cilia of multiple sensory neurons elongate in aged *osm-6* mutants. Since the longer cilia in aged *osm-6* mutants were not full-length (Figs 1B, 1C and 2A–2D, S1 Fig), we asked whether only the middle ciliary segments comprised of doublet microtubules elongate, or whether the elongated cilia also contain singlet microtubules characteristic of ciliary distal segments. As in wild-type animals, we observed distal singlets in a subset of cilia in 7d old *osm-6* mutants (Fig 2E, S3A–S3C Fig), indicating that both middle and distal segments recover upon aging. Together, these results indicate that the cilia of multiple sensory neurons in IFT mutants exhibit AdCR, and that the axonemal ultrastructures of these elongated cilia resemble those of wild-type cilia.

### Cilia-dependent sensory functions are partially restored in aged IFT mutants

**Fig 3:**
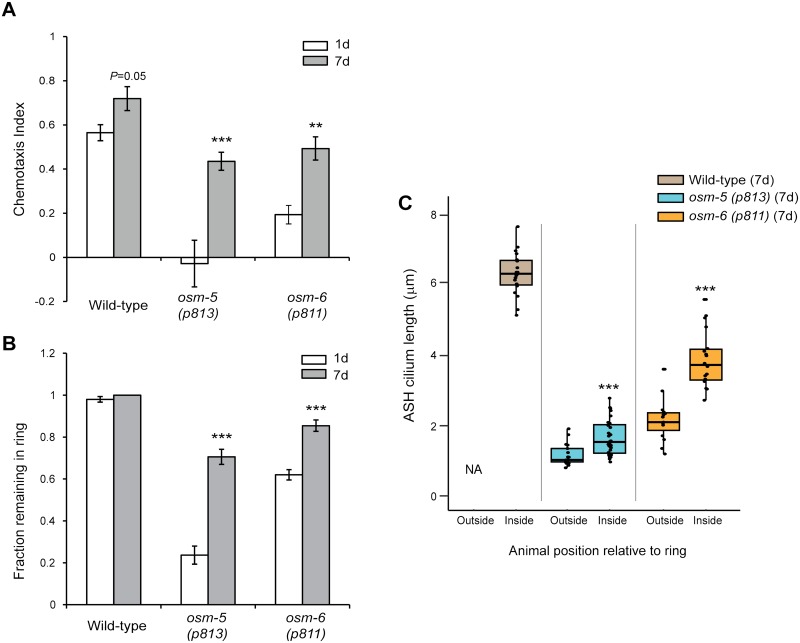
Cilia-dependent sensory behaviors are improved in aged IFT-B mutants. **(A)** Chemotaxis responses of 1d and 7d old animals of the indicated genotypes to a point source of bacteria (see Materials and Methods). Positive chemotaxis indices indicate attraction. ** and *** indicate different from 1d within a genotype at *P*<0.01 and 0.001, respectively (Kruskal-Wallis non parametric test). Error bars are SEM. n>200 animals each from 8 independent assays. **(B)** Fraction of animals of the indicated genotypes and ages that remain within a ring of 8M glycerol after 2 minutes. Error bars are SEM. *** indicate different from 1d at *P*< 0.001 within a genotype (Kruskal-Wallis non-parametric test). n>100 animals each from 10 independent assays. **(C)** Length of ASH cilia in 7d old wild-type, *osm-5(p813)* and *osm-6(p811)* animals that remained within (inside), or escaped (outside), a ring of 8M glycerol after 2 minutes. Horizontal lines indicate 25^th^, 50^th^ and 75^th^ percentiles. *** indicates different from 1d within a genotype at *P*< 0.001 (Wilcoxon Mann-Whitney U test). n≥15 animals for each condition.

We next examined whether in addition to partially restoring cilia morphology, AdCR restores ciliary function and sensory responsiveness of the affected neurons. A subset of ciliated sensory neurons responds to environmental chemical stimuli including volatile and aqueous chemicals produced by the bacterial food source of *C*. *elegans*, and drives attraction or avoidance behaviors [45–47]. Many IFT gene mutants exhibit strong defects in attraction to, or avoidance of, subsets of chemicals [17–19], indicating that intact cilia are essential for chemosensation by many sensory neurons. We first compared the ability of 1d and 7d old wild-type and IFT mutants to be attracted to a point source of live bacteria. We found that while wild-type animals were robustly attracted regardless of age, 1d old *osm-5(p813)* and *osm-6(p811)* mutants exhibited weak or no attraction (Fig 3A). However, attraction to bacteria was dramatically improved in 7d old *osm-5(p813)* and *osm-6(p811)* animals (Fig 3A), implying that AdCR may contribute to improved sensory responsiveness. Decreased chemosensory responses in 1d old *osm* mutant animals were not simply due to movement defects since the velocities of 1d and 7d old *osm-5* and *osm-6* mutants were inversely correlated with their chemoattraction behaviors (S1 Table). We could not examine chemotaxis behaviors of *osm-5(ok451)* mutants since these animals exhibited impaired locomotion due to unknown reasons.

To further correlate AdCR with improved chemosensation, we examined the ability of wild-type and *osm-5* and *osm-6* mutants to avoid solutions of high osmolarity. Osmotic avoidance behavior is mediated by the ASH sensory neurons [48], and IFT gene mutants exhibit strong defects in this avoidance response [17]. As expected, while nearly 100% of wild-type animals placed within a ring of 8M glycerol remained within the ring regardless of age, many 1d old *osm-5(p813)* and *osm-6(p811)* mutants escaped the ring within 2 mins (Fig 3B), consistent with impaired ASH sensory functions in these mutants. As in the case of bacterial chemosensation, 7d old *osm-5* and *osm-6* mutants exhibited improved osmotic avoidance, such that a significantly larger number of animals remained within the ring (Fig 3B).

Improved chemosensory responses in aged IFT mutants could arise due to physiological changes unrelated to AdCR. To address this issue, we next correlated ASH cilia length with osmotic avoidance behavior in 7d old *osm-5(p813)* and *osm-6(p811)* mutants. We found that ASH cilia of both *osm-5* and *osm-6* mutant animals that escaped the ring were on average significantly shorter than those of animals that remained within the ring after 2 mins (Fig 3C). Together, these results suggest that AdCR partially restores sensory cilia function.

### IFT motors are essential for AdCR

**Fig 4:**
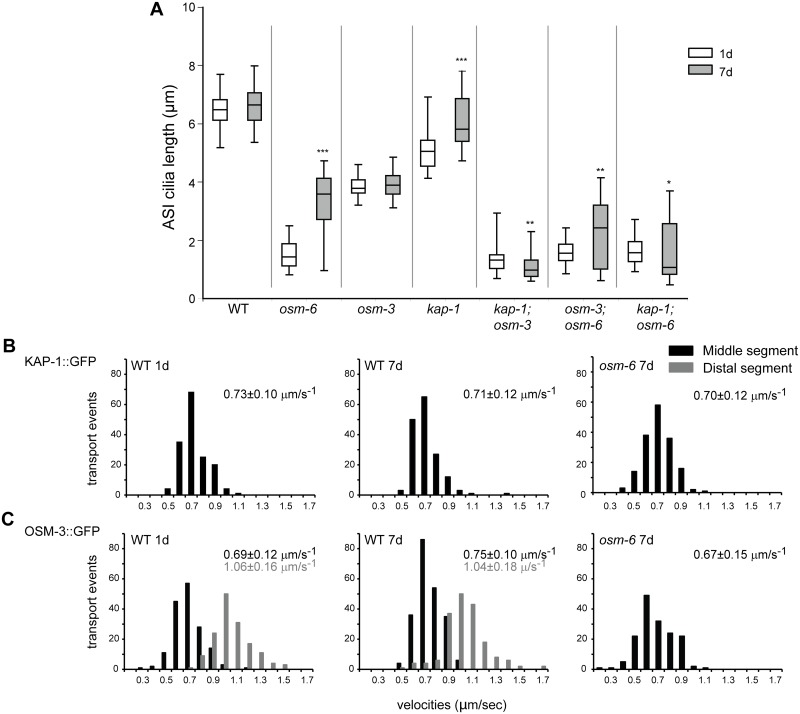
IFT motor proteins are necessary for age-dependent cilia recovery in IFT mutants. **(A)** Quantification of ASI cilia length in 1d and 7d old animals of the indicated genotypes. ASI cilia were visualized via expression of *str-3*p::*srg-36*::*gfp*. Alleles used in the double mutant strains were *osm-6(p811)*, *kap-1(ok676)* and *osm-3(p802)*. Horizontal lines indicate 25^th^, 50^th^ and 75^th^ percentiles; bars indicate 5^th^ and 95^th^ percentiles. *, ** and *** indicate different from 1d within a genotype at *P*<0.05, 0.01 and 0.001, respectively (Wilcoxon Mann-Whitney U test). n>30 for each; ≥3 independent experiments. **(B)** Histograms of KAP-1::GFP and **(C)** OSM-3::GFP anterograde velocities in the ASH/ASI cilia of 1d and 7d old wild-type or *osm-6(p811)* mutants. *kap-1*:*gfp* and *osm-3*::*gfp* were expressed under the *sra-6* promoter. IFT could not be reliably quantified in short cilia in 1d old *osm-6* mutants. Anterograde velocities in the middle and distal segments are indicated by black and gray bars, respectively; average velocities are indicated at top right in each panel in corresponding colors. See S2 Table for statistical analyses.

To begin to explore the mechanisms underlying AdCR, we first asked whether IFT motors are necessary for this process. The middle segments of channel cilia, including ASI cilia, are built via the cooperative and redundant actions of the heterotrimeric kinesin-II (comprised of *klp-11*, *kap-1* and *klp-20*-encoded proteins) and homodimeric OSM-3 kinesin-2 motors, whereas the distal segment requires OSM-3 function alone [49] (Fig 1A). The *osm-3(p802)* allele is predicted to encode a protein that lacks the motor stalk and tail domains thereby likely abrogating interactions between OSM-3 and IFT particles [49]. The distal segments of ASI cilia are absent in *osm-3(p802)* mutants [14], and we found that these shortened cilia did not elongate regardless of age (Fig 4A). Although ASI cilia were also unexpectedly shorter in 1d old *kap-1(ok676)* putative null mutants, these cilia elongated in 7d old animals, likely via OSM-3 function (Fig 4A). Consistent with this hypothesis, the severely truncated ASI cilia in aged *kap-1;osm-3* double mutants did not elongate (Fig 4A).

We next tested whether loss of either motor function affects AdCR in *osm-6* mutants. Although *osm-3(p802)* failed to fully suppress AdCR, loss of *kap-1* suppressed AdCR in *osm-6* mutants (Fig 4A), suggesting that kinesin-II is the primary motor that mediates AdCR in IFT mutants. Thus, while OSM-3 can elongate ASI cilia in aged *kap-1* mutants in the presence of wild-type IFT complexes, this motor is partly dispensable for AdCR in IFT mutants. Consistent with a possible altered function of OSM-3 in aged IFT mutants, kymograph analyses showed that OSM-3 moved anterogradely at a slower rate in the middle segments of ASH/ASI cilia in 7d old *osm-6* mutants as compared to 7d old wild-type animals, whereas the velocity profile of kinesin-II was similar in both genetic backgrounds (Fig 4 and 4C and S2 Table). We conclude that IFT motors, and in particular, kinesin-II, is essential for AdCR in IFT mutants.

### The HSF-1 heat shock factor, but not DAF-16 FOXO, is necessary for AdCR

**Fig 5:**
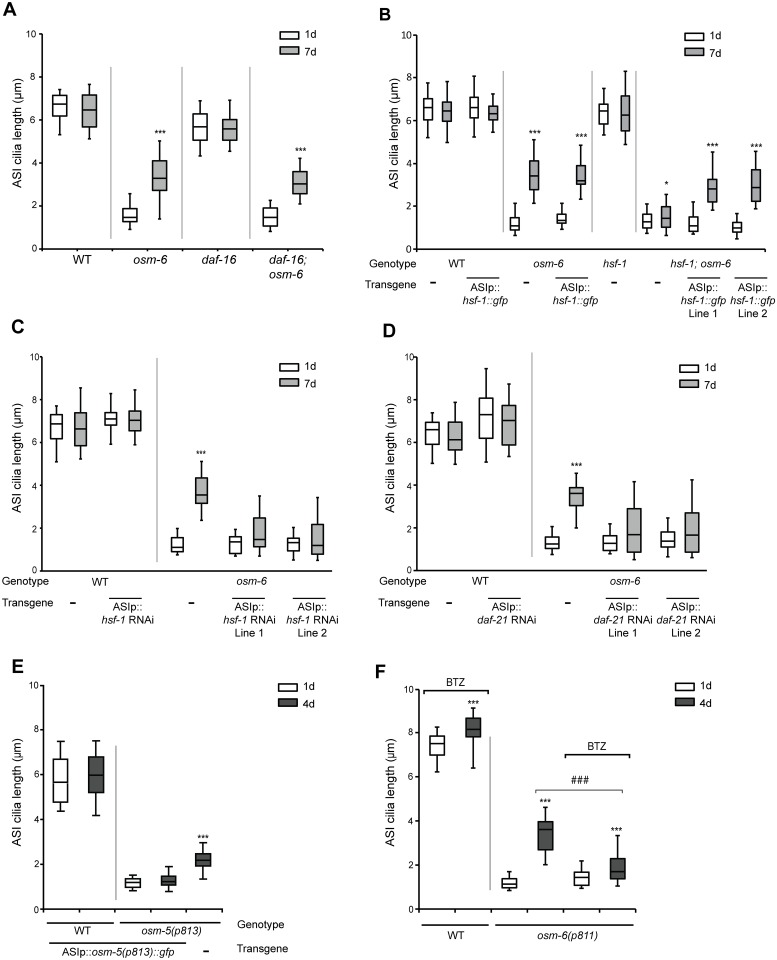
Improved protein quality control mechanisms may underlie age-dependent cilia recovery in IFT mutants. **(A-D)** Quantification of ASI cilia length in animals of the indicated genotypes and adult ages. Alleles used were *osm-6(p811)*, *daf-16(mu86)*, and *hsf-1(sy441ts)*. ASI cilia were visualized via expression of *str-3*p::*srg-36*::*gfp* (A,C,D) or *srg-47*p::*TagRFP* (B). An *hsf-1* cDNA tagged with *gfp*, and *hsf-1* and *daf-21* sense and antisense sequences were expressed in ASI under the *srg-47* promoter. Lines 1 and 2 represent independent transgenic lines. Animals were grown at 20°C (A,C,D) or 25°C (B). Horizontal lines indicate 25^th^, 50^th^ and 75^th^ percentiles; bars indicate 5^th^ and 95^th^ percentiles. * and *** indicate different from 1d within a genotype at *P*<0.05 and 0.001, respectively (Wilcoxon Mann-Whitney U test). n>30 for each; ≥3 independent experiments. **(E)** Quantification of ASI cilia length in 1d and 4d old animals of the indicated genotypes, expressing *srg-47*p::*osm-5(p813)*::*gfp*. ASI cilia were visualized via expression of *srg-47*p::*TagRFP*. Horizontal lines indicate 25^th^, 50^th^ and 75^th^ percentiles; bars indicate 5^th^ and 95^th^ percentiles. *** indicates different from 1d of the same genotype at *P*<0.001 (Wilcoxon Mann-Whitney U test). n>50 each; 3 independent experiments. **(F)** Quantification of ASI cilia length in 1d and 4d old animals of the indicated genotypes. ASI cilia were visualized via expression of *str-3*p::*srg-36*::*gfp*. Animals were grown on 10 μM Bortezomib (BTZ). Horizontal lines indicate 25^th^, 50^th^ and 75^th^ percentiles; bars indicate 5^th^ and 95^th^ percentiles. *** indicates different from 1d within a genotype at *P*<0.001; ^###^ indicates different between the indicated conditions at *P*<0.001 (Wilcoxon Mann-Whitney U test). n>50 each; 3 independent experiments.

We next examined the requirement of signaling pathways implicated in regulating aging in mediating AdCR. In *C*. *elegans* and other organisms, loss or reduction of insulin signaling increases longevity primarily, but not exclusively, via activation of the DAF16/FOXO and HSF-1 transcription factors [50–54]. Both transcription factors in turn regulate the expression of genes including cellular chaperones, which maintain proteostasis and promote longevity [26, 55]. We found that while loss of *daf-16* had no effect on AdCR in *osm-6* mutants (Fig 5A), the *hsf-1(sy441ts)* mutation significantly reduced AdCR in these animals at the restrictive temperature (Fig 5B). Similarly ASI-specific knockdown of *hsf-1* by RNAi abolished AdCR (Fig 5C). ASI-specific overexpression of *gfp*-tagged wild-type *hsf-1* sequences in *osm-6; hsf-1* double mutants rescued AdCR (Fig 5B), although no effects on cilia length were observed upon HSF-1 overexpression in 1d old *osm-6* or *hsf-1;osm-6* mutants (Fig 5B). ASI cilia length was unaffected upon either overexpression or knockdown of *hsf-1* in wild-type animals at any examined age (Fig 5B and 5C). We conclude that HSF-1 acts cell autonomously to regulate AdCR.

HSF1-regulated chaperones such as Hsp90 are upregulated during flagellar regeneration in *Chlamydomonas* [56, 57], although we did not observe transcriptional upregulation of the *daf-21* Hsp90 *C*. *elegans* ortholog upon aging in wild-type or IFT mutant backgrounds (S4 and S4B Fig). Since null mutations in *daf-21* result in larval lethality [58], we tested a requirement for Hsp90 in AdCR by knocking down *daf-21* via cell-specific RNAi in ASI in *osm-6* mutants and quantifying cilia length. As shown in Fig 5D, decreased DAF-21 function in ASI suppressed cilia elongation in *osm-6* mutants. These results suggest that Hsp90 may also be required cell-autonomously for AdCR in hypomorphic IFT-B mutants.

It has previously been shown that although expression of chaperones is not altered upon chronic expression of aggregation-prone proteins in *C*. *elegans*, the extent of aggregation remains HSF1-dependent [59]. We verified that similar to *daf-21*, expression of the heat shock reporter *hsp-16*.*2*p::*gfp* [60, 61] was also unaltered in aged wild-type or *osm-6* animals (S4C Fig). To mimic conditions of chronic stress potentially experienced by aged IFT mutants, we asked whether exposure of 1d old *osm-6* mutants to repeated or prolonged heat stress is sufficient to induce AdCR. We subjected wild-type and *osm-6* L3-L4 larvae to repeated acute heat shock (3 repeated exposures to 34°C for 15 min, interspersed with 15 min recovery at 20°C), or mild prolonged heat shock (28°C for 24°h), and quantified ASI cilia lengths in 1d old adults. However, cilia lengths were unaltered under either heat shock regime (S4D Fig), indicating that exposure to prolonged heat stress is not sufficient to induce AdCR in young animals. Together, these results indicate that HSF-1 and Hsp90 are required for AdCR, but that expression of chaperone proteins in ciliated sensory neurons is unaffected upon aging in *osm-6* mutants.

### Overexpression of mutant IFT protein inhibits AdCR

Since AdCR only occurs in animals carrying hypomorphic alleles of IFT genes, we considered the possibility that AdCR is mediated by the accumulation of partially functional IFT proteins as a function of chronological age or due to age-dependent failure of proteostasis [26–29]. This hypothesis predicts that overexpression of the mutant IFT protein will promote cilia recovery in young adult animals, and may further enhance AdCR in aged IFT hypomorphic mutants.

To test this notion, we overexpressed a GFP-tagged *osm-5(p813)* cDNA specifically in ASI in wild-type and *osm-5(p813)* mutants, and examined cilia length in 1d and 4d old animals. A wild-type OSM-5::GFP fusion protein was able to rescue *osm-5* but not *osm-6* mutant phenotypes and localized to cilia when expressed in ASI (S5A Fig), indicating that addition of GFP coding sequences does not alter OSM-5 protein function. Contrary to our prediction, we found that ASI cilia length in 1d old *osm-5(p813)* mutants was unaffected upon overexpression of the mutant OSM-5 protein (Fig 5E). Instead, overexpression of the mutant IFT protein abolished AdCR in 4d old *osm-5(p813)* animals (Fig 5E). No effects on ASI cilia length were observed in a wild-type background (Fig 5E). We could not perform similar experiments with an *osm-6(p811)* encoded protein since this mutation results in the production of multiple alternatively spliced mRNAs (S2B Fig) complicating experimental design. We conclude that overexpression of a mutant OSM-5 protein inhibits AdCR.

### Improved proteostasis mechanisms may be necessary for AdCR

Based on the above observation, we hypothesized that mutant IFT proteins may be toxic, and that removal of these proteins in aged animals permits productive IFT and AdCR. The ubiquitin-proteasome system (UPS) plays a major role in the degradation of misfolded and toxic proteins associated with aging and diseases [31, 32, 62, 63]. The UPS has also been implicated in cilia biology [64, 65]. To test whether UPS activity plays a role in AdCR, we transferred L4 larval stage animals to plates containing the 26S proteasome inhibitor Bortezomib and grew them to adulthood [66]. While Bortezomib treatment had no effect on ASI cilia length in 1d old *osm-6* mutants, growth on this reagent significantly inhibited AdCR in 4d old *osm-6* mutants (Fig 5F). ASI cilia length in 4d old wild-type animals was weakly but significantly increased upon Bortezomib treatment (Fig 5F) for reasons that are currently unclear. Moreover, levels of Ub-G76V::GFP—an inverse reporter of UPS activity [67–71]—were significantly decreased in ASI neurons of 4d old wild-type and *osm-6* mutants as compared to 1d old animals (S5B Fig), suggesting that UPS activity is upregulated in these neurons during early aging. We infer that increased UPS activity in ciliated sensory neurons during early aging contributes to AdCR.

Since AdCR requires both HSF-1 and UPS activity, we investigated whether AdCR is correlated with improved proteostasis in ciliated sensory neurons. Decreased aggregation of the human SOD1(G85R) protein has been shown to correlate with improved protein quality control in *C*. *elegans* neurons [72–74]. As reported previously [73, 75], expression of SOD1(G85R)::YFP resulted in the formation of aggregates of heterogeneous sizes with large and small aggregates in body wall muscle and ASI neurons, respectively (S5C Fig). While aggregates in muscle did not appear to be grossly affected by age or genetic background, the number of small aggregates in ASI decreased in both 4d old wild-type and *osm-6* animals (S5D Fig). This reduction in aggregate number was not correlated with reduced ASI promoter activity in aged animals (S5E Fig). These results suggest that improved proteostasis may contribute to AdCR.

## Discussion

We report that aging partly suppresses the severe cilia structural defects of IFT hypomorphic mutants in *C*. *elegans*. Remarkably, AdCR correlates with significant recovery of cilia-dependent sensory behaviors; aged IFT mutants exhibit markedly improved chemosensory responses to both attractive and noxious cues. This result is surprising *a priori* since many IFT gene mutants were originally identified on the basis of their severe chemosensory defects [17–19]. However, the majority of behavioral screens were likely performed using 1-2d old young adult animals which exhibit highly defective cilia, thereby enabling the isolation of these chemotaxis-defective IFT mutants. While structural recovery is observed by 4d of aging in animals grown at 20°C, cilia of IFT mutants are again truncated during late stages of aging, indicating that AdCR is a transient process.

AdCR is dependent on IFT. This conclusion is based on several observations. *First*, kinesin-II is essential for this process. In wild-type animals, kinesin-II and OSM-3 act redundantly to build the middle segments of the cilia of a subset of sensory neurons including ASI [49]. However, OSM-3 alone cannot extend the middle segments in aged *osm-6* mutants, suggesting that OSM-3 functions are altered under these conditions. Consistent with this hypothesis, OSM-3 anterograde velocity is decreased in the middle segments of ASI cilia in 7d old *osm-6* mutants as compared to its velocity in wild-type ASI cilia in animals of the same age. AdCR is also abolished in *osm-3;kap-1* double mutants. *Second*, the structural recovery is observed in diverse cilia types during early aging, suggesting that AdCR is mediated by a process that is common to all cilia. *Third*, AdCR is only observed in animals carrying hypomorphic, but not null, alleles of IFT genes, indicating that partial IFT protein function is necessary for this process. Together, these results suggest that AdCR is mediated by partial restoration of IFT function in hypomorphic IFT mutants.

HSF1/Hsp90 buffer the effects of partial loss-of-function mutations [76–78]. However, it is unlikely that simple genetic buffering via upregulation of HSF1/Hsp90 during aging is sufficient for AdCR since neither overexpression of HSF1 nor induction of the heat shock response in 1d old *osm-6* mutants is sufficient to suppress their ciliary defects. Instead, we speculate that in younger animals, expression of a partly functional IFT protein in the absence of the wild-type protein disrupts IFT complex function [35, 36, 79, 80]. Reduced levels of mutant protein in aged animals via increased UPS activity, coupled with chaperone-mediated stabilization of the complex or folding intermediates enables productive IFT and AdCR in IFT hypomorphic mutant animals (S6 Fig). HSF-1/Hsp90 and UPS may also indirectly affect IFT to improve ciliogenesis. Hsp90 has been suggested to facilitate tubulin polymerization [81, 82]; increased tubulin assembly mediated by Hsp90 may also promote productive IFT [83] in aged IFT hypomorphic mutant backgrounds.

A positive effect of aging on cilia structure and function is unexpected given the association of aging with a decline in cellular functions. However, proteasome function may be regulated in a tissue-specific manner as a function of age [26, 28, 70, 84, 85], indicating that the function of this proteolytic complex is under both local and global regulation. Interestingly, the time period during which AdCR is exhibited in *C*. *elegans* coincides approximately with their reproductive period. Since cilia are essential for the functions of sensory neurons, and sensory neuron functions in turn are required for behaviors such as egg-laying and mate-finding in *C*. *elegans* [86–90], we speculate that AdCR may represent a homeostatic mechanism to maintain sensory cilia function and reproductive fitness under specific conditions.

Similar mechanisms may operate in other organisms to maintain cilia function in IFT mutants. In *Tetrahymena* and *Chlamydomonas*, flagellar defects due to partial loss of IFT proteins can be bypassed in some suppressor strains under conditions of oxygen deprivation [80, 91–93], and it has been suggested that a stress-induced chaperone mechanism stabilizes the IFT-B complex to permit cilia function under these conditions [92]. Moreover, Hsp90 is localized to cilia, and regulates cilia stability in response to stress in mammalian cells [94–96]. Our observations indicate that while IFT protein function is essential for ciliogenesis, compromised IFT complex function can be partly bypassed during early stages of adult aging or under other conditions of stress to promote cilia lengthening. We propose that therapies relieving proteostatic stress may represent a promising avenue for targeting ciliopathies arising from specific mutations in IFT genes.

## Materials and Methods

### Growth of *C*. *elegans*

Worms were grown on *E*. *coli* OP50 bacteria using standard procedures. Double-mutants strains were generated using standard genetic methods, and the presence of the desired alleles was verified by PCR-based genotyping and/or sequencing. Co-injection markers for transgenic strains were *unc-122*p::*gfp* or *unc-122*p::*dsRed* injected at 30 ng/μl and 50 ng/μl, respectively. A complete list of strains is provided in S3 Table.

To age animals, well-fed animals were maintained for at least two generations before analyses. To obtain worms of a specific age, animals were selected at the L4 stage and maintained until the required day of adulthood. Animals were transferred daily to new plates to remove progeny. All animals were grown at 20°C, unless indicated otherwise. For Bortezomib treatment, L4 larval stage worms were grown to adulthood on plates supplemented with 10 μM Bortezomib.

### Molecular biology

0.7 or 1.0 kb of *srg-47* upstream regulatory sequences were used to drive expression of fluorescent reporters, or cDNAs with or without tagged reporter sequences, specifically in ASI. *srg-36*::*gfp* coding sequences were driven under *str-3* upstream regulatory sequences in ASI [38]. The *hsf-1*::*gfp* containing plasmid was a gift from Ao-Lin Hsu (University of Michigan). The *srg-47*p::*osm-6(p811)*::*gfp*::*SL2*::*mCherry* construct was generated by introducing *osm-6* genomic sequences amplified from the PR811 strain (S3 Table) into a construct containing *SL2*::*mCherry* (gift of Cori Bargmann) driven under *srg-47* upstream regulatory sequences. The *p813* mutation was introduced by deleting 3’ sequences in an *osm-5* cDNA cloned under *srg-47* promoter sequences. The G85R mutation was introduced by site-directed mutagenesis into a plasmid containing *unc-54*p::SOD1::YFP coding sequences (kind gift of R. Morimoto). SOD1(G85R)::YFP and Ub-G76V::GFP encoding sequences (plasmid #11941—Addgene) were inserted under *srg-47* regulatory sequences in a worm expression vector. All constructs were verified by sequencing.

*osm-6(p811)* encoded transcripts were identified from mRNA pools isolated from 1d and 4d old animals by reverse transcription, followed by amplification, cloning and sequencing. RNAi constructs were generated as described previously [97] by fusing sense and antisense products obtained from amplifying exon 1 and exon 4 sequences from *hsf-1* and *daf-21*, respectively, to *srg-47* upstream regulatory sequences. Sense and antisense fusion products were subcloned into the pGEM vector (Promega), amplified, and injected at 100 ng/μl each. Primers used for RNAi constructs were the following (5’-3’):

***hsf-1*:**
AC179: GATTCCCTGTTGGCTGCATTTTACGTTTTAATTCGAAGAAAAG
AC180: TGCGTATTTGGAGACCTTGGTAGGGTTTTAATTCGAAGAAAAG
AC182: CCCTACCAAGGTCTCCAAATACGCA
AC183: ATGCAGCCAACAGGGAATCAAA
AC184: CAAGGTCTCCAAATACGCATTATTC
AC185: CGTAAAATGCAGCCAACAGGGAATC

***daf-21*:**
AC186: CGACACGATCACGAAGTGTCCTGAAATTTTAATTCGAAGAAAAG
AC187: GCATGGAGGAGGTCGACTAAACATCCTTTTAATTCGAAGAAAAG
AC188: GGATGTTTAGTCGACCTCCTCCATGC
AC189: GACACTTCGTGATCGTGTCGAGG
AC190: TTAGTCGACCTCCTCCATGCGG
AC191: TTTCAGGACACTTCGTGATCGTGTCG

***Common to both*:**
AC178: CCTGCAGGGAACCATCGATGAAAAACGC
AC181: GAACCATCGATGAAAAACGCTAG

### Microscopy

To perform cilia length measurements, animals were anesthetized with 10 mM tetramisole hydrochloride (Sigma) or sodium azide, mounted on 10% agarose pads on microscope slides, and examined on an inverted spinning disk confocal microscope using a 100X objective (Zeiss Axio Observer with a Yokogawa CSU-22 spinning disk confocal head), or on a Zeiss Axio Imager 2 epifluorescent microscope using a 63X objective. Optical sections were acquired at 0.1 or 0.2-μm intervals and images were *z*-projected at maximum intensity. Cilia length was measured using ImageJ (National Institutes of Health). For optimal visualization of cilia, images were linear adjusted for brightness and contrast using ImageJ (NIH).

IFT analyses were performed as described previously [98]. In brief, movies of mobile GFP particles in the cilia were acquired on a spinning disk confocal microscope for 1–2 mins with a 300 ms exposure time. Kymograph analyses were performed using the Multiple Kymograph plugin in ImageJ (NIH). Average velocities were calculated using data from at least 3 independent experiments. KAP-1::GFP and OSM-3::GFP movement in wild-type and *osm-6* mutants of the same age were imaged together in individual experiments.

For quantification of Ub-G76V::GFP fluorescence levels relative to TagRFP expressed in the same cells, animals were imaged on a spinning disk microscope using a 63X objective. Images were obtained at 0.5 μm intervals, *z*-projected at maximum intensities, and fluorescence quantification performed using ImageJ. Images were acquired with a 100 ms exposure for both fluorophores ensuring that fluorescence levels were not saturated. Mean GFP intensities in the region of interest were normalized to mean RFP intensities to obtain the normalized Ub-G76V::GFP fluorescence values.

*srg-47*p::*TagRFP* levels in ASI were examined in animals co-expressing *srg-47*p::*Ub-G76V*::*gfp* and *srg-47*p::*TagRFP*. Animals were imaged using a 63X objective on a spinning disc confocal microscope. For quantification of fluorescence levels, the ASI cell bodies were marked manually, and quantification was performed on maximum intensity *z*-projected images using ImageJ (NIH).

### Transmission electron microscopy

Adult animals of the desired ages were fixed, sectioned and imaged essentially as previously described [99], with the exception that worms were fixed overnight at 4°C in 2.5% gluteraldehyde, 1% paraformaldehyde in Sørensen phosphate buffer (0.133M, pH 7.2). Serial ultrathin sections of 80 nm were examined on an electron microscope (Tecnai Twin), and images were recorded using a MegaView 2 digital recording system (Olympus).

### Single molecule FISH (smFISH)

smFISH probes were designed against *daf-21* sequences utilizing the Stellaris FISH Probe Designer (Biosearch Technologies, Inc; (www.biosearchtech/com/stellarisdesigner). Probe sets of 44 probes of 22 nucleotides each labeled with TAMRA dye (Biosearch Technologies, Inc.) were used. At least 10–20 1d and 4d old adult animals per strain were fixed using 4% paraformaldehyde and resuspended in 70% ethanol at 4°C for approximately 24 hours. Samples were then hybridized with the *daf-21* Stellaris FISH Probe set following the manufacturer’s instructions (www.biosearchtech.com/stellarisprotocols). For quantification of puncta, images were acquired on an Axio Observer A1 inverted microscope (Zeiss) using a 63X oil objective and a digital CCD camera (Orca-R2 C10600-10B, Hamamatsu). All samples were imaged under identical settings. Mean pixel intensities in the regions containing ASH/ASI neuronal cell bodies were measured using ImageJ (NIH).

### Behavioral measurements

#### Quantification of velocities

To measure velocities, 20 animals of the appropriate adult ages were picked onto a 10 mm agar plate without food and placed on an aluminum plate maintained at 20°C. Animals were allowed to move for 5 mins, and movement was then recorded for 30 mins at a rate of 1 Hz using a USB 3.0 camera (PL-D721, PixeLINK) and the Capture OEM image capture application (PixeLINK). Velocities were calculated using custom written scripts in MATLAB.

#### Bacterial chemotaxis assay

25 μl of a saturated *E*. *coli* culture (OD– 0.9–1.0) was spotted at one end of a chemotaxis assay plate (www.wormbook.org) and allowed to dry for 2 hours prior to the assay. At the start of the assay, 1 μl of sodium azide was placed at the bacterial source as well as at a control spot at the opposite end of the assay plate. 25–75 animals of the appropriate adult ages were then transferred to the center of the assay plate and allowed to move freely for an hour. The chemotaxis index was calculated as (number of worms at the bacteria—number of worms at the control)/total number of worms on the plate.

#### Osmotic avoidance assay

10 adult animals were placed in the center of an 8M glycerol ring with a diameter of 1.5 cm on a 60 mm agar plate as described (www.wormbook.org). The number of animals remaining within the ring, and/or animals outside the ring were counted after 2 mins. To correlate cilia lengths with osmotic avoidance behaviors, animals within or outside the ring after 2 mins were transferred to a slide and ASH cilia lengths were quantified as described above.

### Statistical analyses

All statistical analyses were performed using the SPSS 21 statistical analyses software (IBM). The Wilcoxon Mann-Whitney U or Kruskal-Wallis nonparametric tests were used for data with non-normal distributions.

## Supporting Information

S1 Fig ASI cilia elongate in aged IFT mutants. **A-B)** Representative images of the ASI cilium (left) and quantification of ASI cilia length (right) in animals of the indicated genotypes and ages. Arrowheads mark the cilium base. Cilia in A were visualized via expression of *str-3*p::*srg-36*::*gfp*. Cilia in B were visualized via expression of *srg-47*p::*TagRFP*; the ciliary base was marked by localization of MKS-5::GFP expressed under the *srg-47* promoter. *** different from 1d within a genotype at *P*<0.001 (Wilcoxon Mann-Whitney U test). Anterior is at top. Scale bar: 5 μm. n>30 for each; ≥3 independent experiments.
(TIF)

S2 Fig Canonical IFT gene mutations are hypomorphic. **A)** Genomic structures of the indicated genes with the locations and nature of lesions in the alleles used in this work. The site of insertion of *gfp* in the construct examined in **C** is also shown. The exact molecular identity of the *osm-1(p816)* mutation was determined by sequencing. Boxes shaded in black and gray indicate untranslated sequences and sequences predicted to encode TPR repeats (OSM-5, OSM-1), respectively.
**B)** Structures of cDNAs reverse transcribed from mRNA isolated from two independent populations each of 1d and 4d old *osm-6(p811)* animals. Percentages of identified cDNAs corresponding to each structure are shown. Red arrowheads indicate predicted termination codons. Blue arrows indicate location of a secondary in-frame ATG.
**C)** The protein encoded by the *osm-6(p811)* allele is expressed and localized to cilia. Representative images of ASI cilia in animals expressing the *osm-6(p811)*::*gfp*::SL2::*mCherry* bicistronic operon driven under the *srg-47* promoter. *gfp* coding sequences were inserted in frame prior to the stop codon in *osm-6* sequences containing the *p811* mutation as shown in **A.** Images from two independent transgenic lines are shown. Anterior is at top. Arrowheads mark cilia base. Scale bar: 5 μm.
(TIF)

S3 Fig Transmission electron microscopy of amphid channel sensory pores in wild-type and *osm-6(m533)* mutant worms of the indicated ages. **A)** Serial cross section images of the entire nose tip at low magnification (left) and amphid pores at high magnification (right; boxed regions in images at left). Boxed numbers denote proximal positioning of section relative to distal-most first section. Bars; 2 μm (images at left), 200 nm (images at right). A subset of these images is also shown in Fig 2E.
**B**) Additional images of the cilia endings in 1d and 7d old *osm-6* adults. Bars; 100 nm (large panels), 50 nm (small panels).
**C)** Schematics summarizing amphid pore ultrastructure. Wild type pores possess 10 ciliary axonemes (only 3 shown), each with distal segment (DS; singlet microtubules), middle segment (MS; doublet microtubules), transition zone (TZ) and periciliary membrane (PCMC) subcompartments. Numbers indicate the section positions shown in **A, B,** and **D**.
**D)** Quantification of axoneme numbers in the distal and proximal pores of wild-type and *osm-6(m533)* animals. Section positions are as indicated in **C**. n = 1 animal each for 1d and 7d old wild-type; 2 animals each for 1d and 7d old *osm-6(m533)*.
(TIF)

S4 Fig Expression of *daf-21* and *hsp-16*.*2* is not upregulated in aged IFT mutants. **A)** Representative maximum projection confocal images of heads of 1d and 4d old adult animals of the indicated genotypes. *daf-21* mRNA expression was detected by single molecule fluorescent *in situ* hybridization (smFISH). Scale bar: 25 μm. Both wild-type and *osm-5* strains contain stably integrated copies of an *sra-6*p::*gfp* transgene. Anterior is at left/bottom in all images.
**B)** Quantification of *daf-21* mRNA fluorescence across the ASH and ASI cell bodies. AU—arbitrary fluorescence units per unit area of neuronal cell bodies. n = 13–24 animals each. Errors are SD.
**C)** Representative images of *hsp-16*.*2*p::*gfp* [100] in the head regions of 1d and 4d old animals of the indicated genotypes. Numbers in top right corner indicate the percentage of animals exhibiting the shown pattern. n>20 animals each. Anterior is at left. Scale bar: 10 μm.
**D)** ASI cilia lengths visualized via expression of *str-3*p::*srg-36*::*gfp* in wild-type and *osm-6(p811)* 1d old adults grown continuously at 20°C (no heat shock), subjected to heat shock at 34°C for 15 mins with intervals of 15 mins at 15°C (repeated heat shock), or 28°C for 24 hrs (mild prolonged heat shock). Heat shock was performed in L3-L4 larval stage animals. Wild-type (no heat shock) data are shown for comparison from an independent experiment and were not analyzed concurrently (indicated in gray). Horizontal lines indicate 25^th^, 50^th^ and 75^th^ percentiles; bars indicate 5^th^ and 95^th^ percentiles. n>25 for each; ≥2 independent experiments.
(TIF)

S5 Fig Proteostasis is improved in ciliated sensory neurons in aged animals. **A)** Expression of *osm-5*::*gfp* under the *srg-47* promoter sequences restores ASI cilia length in *osm-5(ok451)* but not *osm-6(p811)* mutants. White and yellow arrowheads indicate cilia base and cilia axoneme, respectively. Magnified images of cilia are shown in the insets. Anterior is at top. Scale bar: 10 μm; insets– 5 μm.
**B)** Ratio of fluorescence levels of Ub-G76V::GFP to TagRFP in the ASI soma of 1d and 4d old animals of the indicated genotypes grown at 20°C (left) and 25°C (right). Both transgenes were expressed under *srg-47* promoter sequences. *** indicates different from 1d of the same genotype at *P*<0.001 (Wilcoxon Mann-Whitney U test). n>45 each; 3 independent experiments.
**C)** Large and small aggregates of SOD1(G85R)::YFP protein in body wall muscle and ASI soma in 1d and 4d old wild-type and *osm-6(p811)* mutants. Expression was driven under the *unc-54* (muscle) and *srg-47* promoters (ASI). Large and small SOD1(G85R)::YFP aggregates were defined as puncta that were >3 μm and <1 μm in diameter, respectively. Scale bar: 10 μm.
**(D)** Average number of small and medium sized aggregates of SOD1(G85R)::YFP in the ASI soma of 1d and 4d old animals of the indicated genotypes. Expression in ASI was driven under the *srg-47* promoter. Animals also expressed SOD1(G85R)::YFP under muscle specific *unc-54* regulatory sequences (see S5C Fig). Small and medium SOD1(G85R)::YFP aggregates were defined as puncta that were <1 μm and between 1–3 μm in diameter, respectively. ^##^ and ^#^ indicate different from 1d within a genotype at *P*<0.005 and 0.05, respectively (Wilcoxon Mann-Whitney U test). n>30 each; 3 independent experiments.
**E)** Quantification of *srg-47*p::TagRFP levels in ASI neurons of wild-type and *osm-6(p811)* animals of the indicated ages. n>45 neurons each; 3 independent experiments. AU—arbitrary fluorescence units.
(TIF)

S6 Fig Model for the role of proteasome and HSF1 activity in mediating AdCR. In larvae/young adults, accumulation of a partially functional IFT-B protein disrupts IFT resulting in a truncated sensory cilium and defective chemosensation. In middle-aged adults, degradation of mutant IFT proteins by increased proteasome activity, and improved protein folding or stabilization of the IFT complex by HSF1/chaperones, may lead to partially functional IFT, AdCR, and improved chemosensory behaviors. The function of OSM-3 may be also altered in aged IFT mutant animals. See text for additional details.
(TIF)

S1 Table Average velocities of aged animals. (DOCX)

S2 Table Anterograde IFT velocities in ASH/ASI cilia. (DOCX)

S3 Table List of strains used in this work. (DOCX)
