# Supplementary figures and images for: Structural and Functional Recovery of Sensory Cilia in C. elegans IFT Mutants upon Aging
Source: PLoS Genet. 2016 Dec 1;12(12):e1006325. doi: 10.1371/journal.pgen.1006325 (PMC5131903; doi:10.1371/journal.pgen.1006325)

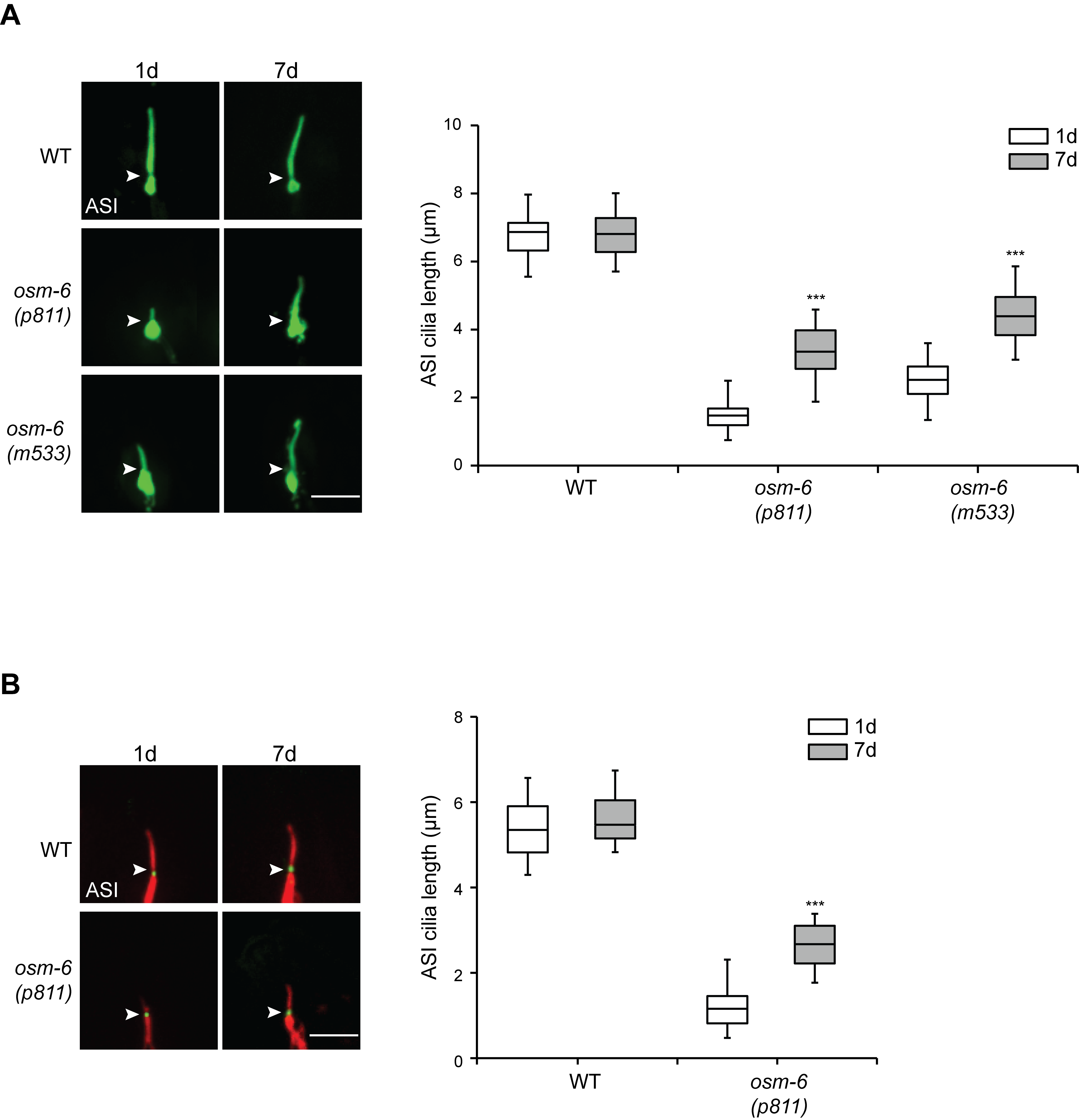

Supplement: S1 Fig — A-B) Representative images of the ASI cilium (left) and quantification of ASI cilia length (right) in animals of the indicated genotypes and ages. Arrowheads mark the cilium base. Cilia in A were visualized via expression of str-3p::srg-36::gfp. Cilia in B were visualized via expression of srg-47p::TagRFP; the ciliary base was marked by localization of MKS-5::GFP expressed under the srg-47 promoter. *** different from 1d within a genotype at P<0.001 (Wilcoxon Mann-Whitney U test). Anterior is at top. Scale bar: 5 μm. n>30 for each; ≥3 independent experiments. (TIF) [file pgen.1006325.s001.tif]

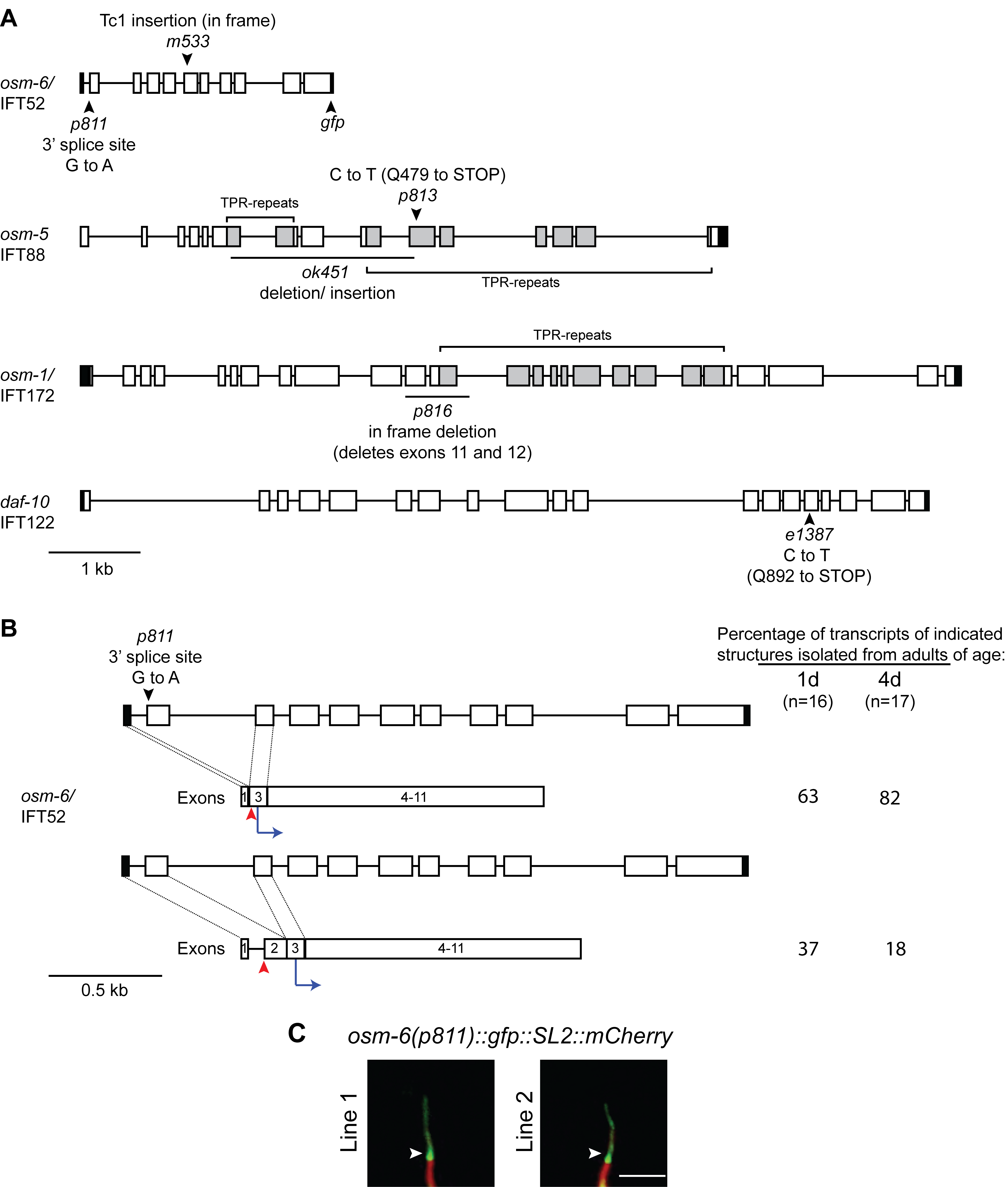

Supplement: S2 Fig — A) Genomic structures of the indicated genes with the locations and nature of lesions in the alleles used in this work. The site of insertion of gfp in the construct examined in C is also shown. The exact molecular identity of the osm-1(p816) mutation was determined by sequencing. Boxes shaded in black and gray indicate untranslated sequences and sequences predicted to encode TPR repeats (OSM-5, OSM-1), respectively. B) Structures of cDNAs reverse transcribed from mRNA isolated from two independent populations each of 1d and 4d old osm-6(p811) animals. Percentages of identified cDNAs corresponding to each structure are shown. Red arrowheads indicate predicted termination codons. Blue arrows indicate location of a secondary in-frame ATG. C) The protein encoded by the osm-6(p811) allele is expressed and localized to cilia. Representative images of ASI cilia in animals expressing the osm-6(p811)::gfp::SL2::mCherry bicistronic operon driven under the srg-47 promoter. gfp coding sequences were inserted in frame prior to the stop codon in osm-6 sequences containing the p811 mutation as shown in A. Images from two independent transgenic lines are shown. Anterior is at top. Arrowheads mark cilia base. Scale bar: 5 μm. (TIF) [file pgen.1006325.s002.tif]

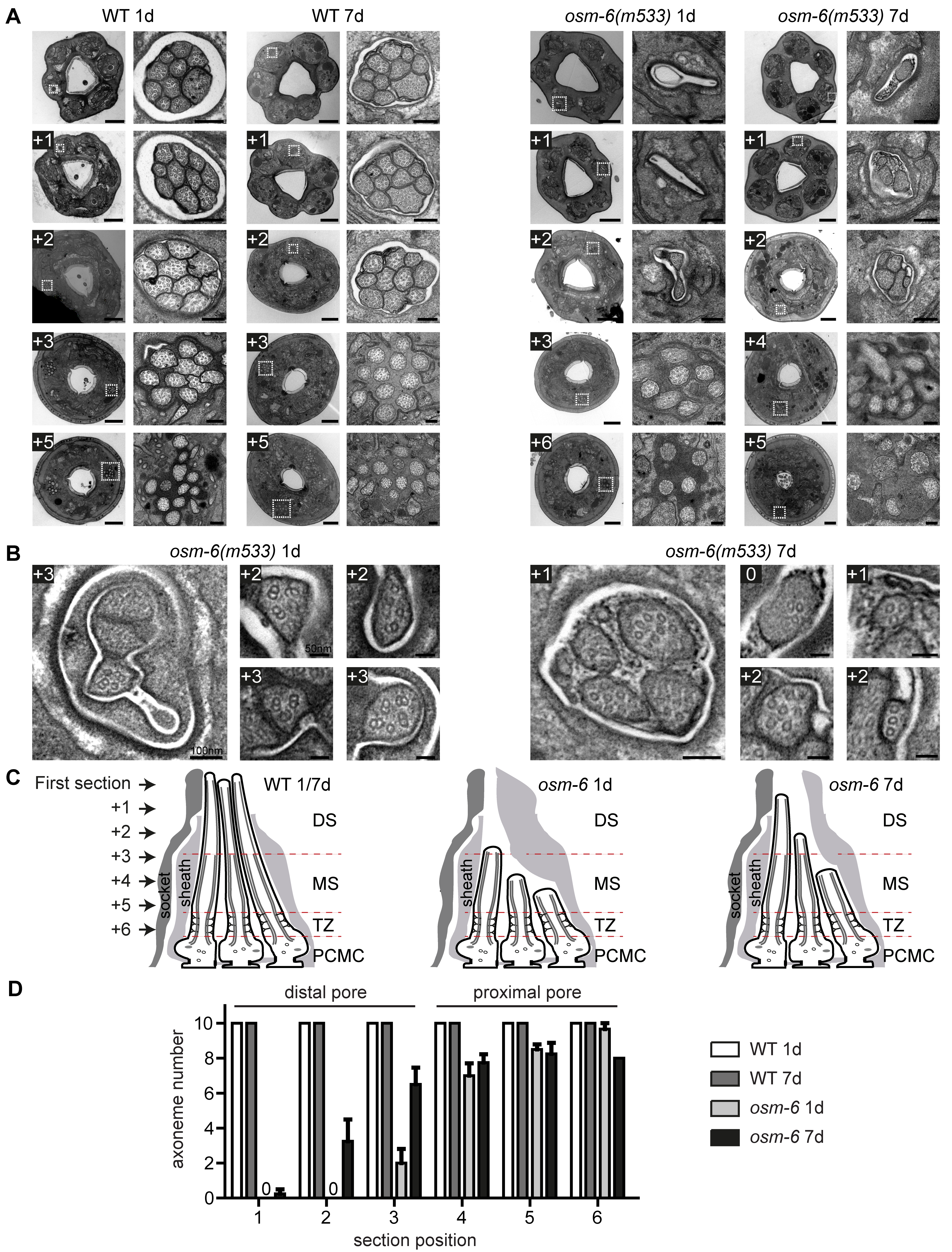

Supplement: S3 Fig — A) Serial cross section images of the entire nose tip at low magnification (left) and amphid pores at high magnification (right; boxed regions in images at left). Boxed numbers denote proximal positioning of section relative to distal-most first section. Bars; 2 μm (images at left), 200 nm (images at right). A subset of these images is also shown in Fig 2E. B) Additional images of the cilia endings in 1d and 7d old osm-6 adults. Bars; 100 nm (large panels), 50 nm (small panels). C) Schematics summarizing amphid pore ultrastructure. Wild type pores possess 10 ciliary axonemes (only 3 shown), each with distal segment (DS; singlet microtubules), middle segment (MS; doublet microtubules), transition zone (TZ) and periciliary membrane (PCMC) subcompartments. Numbers indicate the section positions shown in A, B, and D. D) Quantification of axoneme numbers in the distal and proximal pores of wild-type and osm-6(m533) animals. Section positions are as indicated in C. n = 1 animal each for 1d and 7d old wild-type; 2 animals each for 1d and 7d old osm-6(m533). (TIF) [file pgen.1006325.s003.tif]

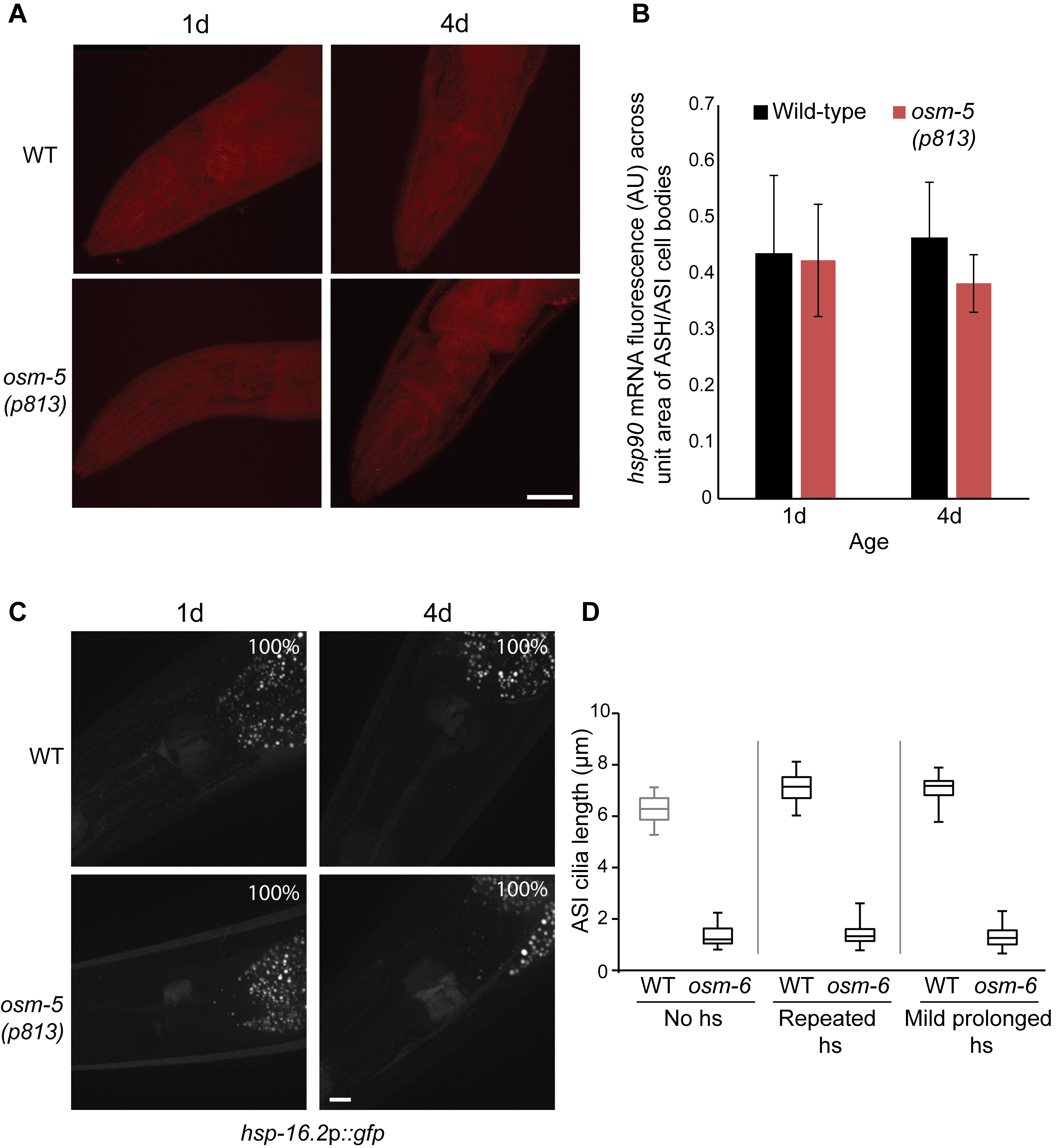

Supplement: S4 Fig — A) Representative maximum projection confocal images of heads of 1d and 4d old adult animals of the indicated genotypes. daf-21 mRNA expression was detected by single molecule fluorescent in situ hybridization (smFISH). Scale bar: 25 μm. Both wild-type and osm-5 strains contain stably integrated copies of an sra-6p::gfp transgene. Anterior is at left/bottom in all images. B) Quantification of daf-21 mRNA fluorescence across the ASH and ASI cell bodies. AU—arbitrary fluorescence units per unit area of neuronal cell bodies. n = 13–24 animals each. Errors are SD. C) Representative images of hsp-16.2p::gfp [100] in the head regions of 1d and 4d old animals of the indicated genotypes. Numbers in top right corner indicate the percentage of animals exhibiting the shown pattern. n>20 animals each. Anterior is at left. Scale bar: 10 μm. D) ASI cilia lengths visualized via expression of str-3p::srg-36::gfp in wild-type and osm-6(p811) 1d old adults grown continuously at 20°C (no heat shock), subjected to heat shock at 34°C for 15 mins with intervals of 15 mins at 15°C (repeated heat shock), or 28°C for 24 hrs (mild prolonged heat shock). Heat shock was performed in L3-L4 larval stage animals. Wild-type (no heat shock) data are shown for comparison from an independent experiment and were not analyzed concurrently (indicated in gray). Horizontal lines indicate 25th, 50th and 75th percentiles; bars indicate 5th and 95th percentiles. n>25 for each; ≥2 independent experiments. (TIF) [file pgen.1006325.s004.tif]

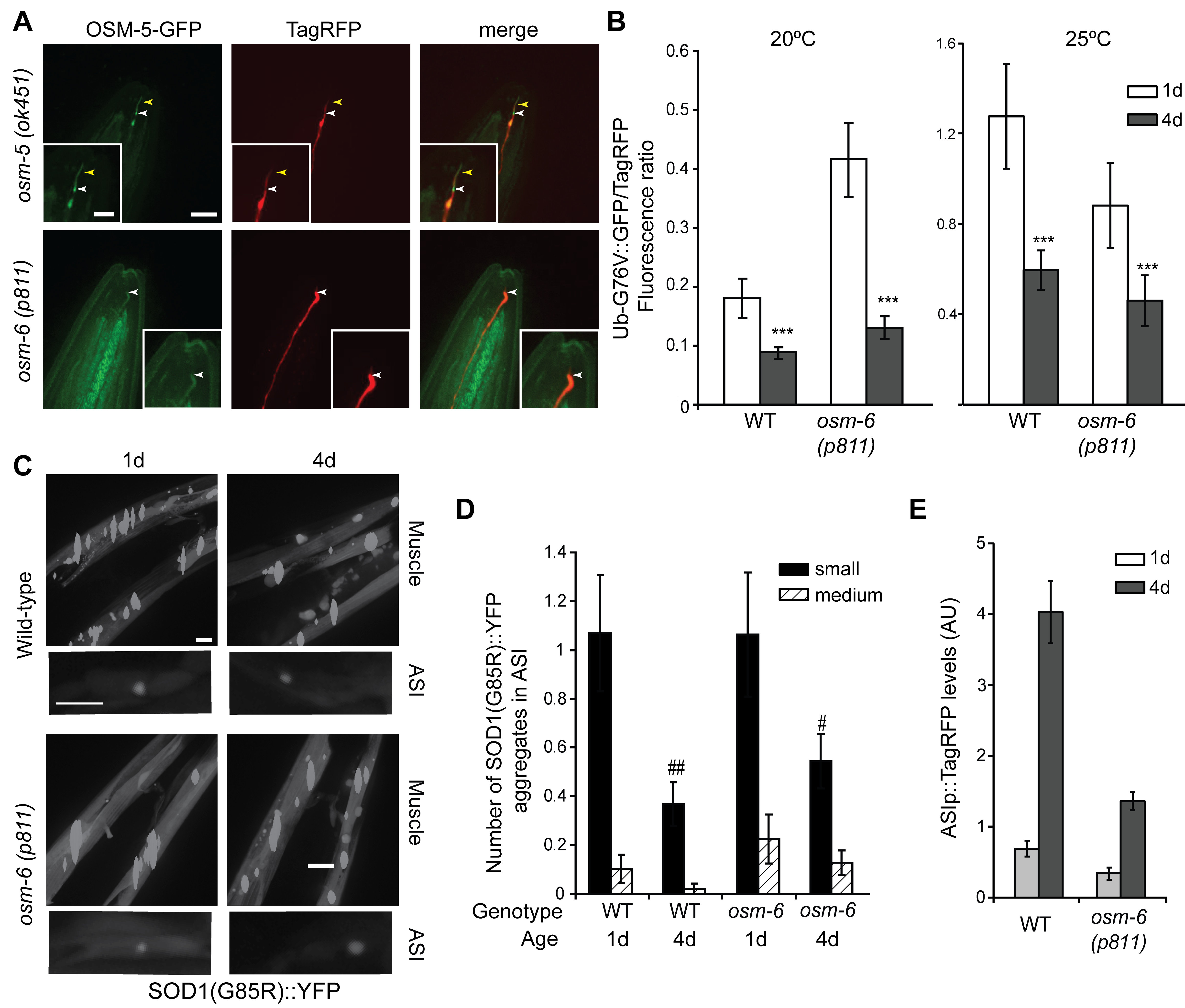

Supplement: S5 Fig — A) Expression of osm-5::gfp under the srg-47 promoter sequences restores ASI cilia length in osm-5(ok451) but not osm-6(p811) mutants. White and yellow arrowheads indicate cilia base and cilia axoneme, respectively. Magnified images of cilia are shown in the insets. Anterior is at top. Scale bar: 10 μm; insets– 5 μm. B) Ratio of fluorescence levels of Ub-G76V::GFP to TagRFP in the ASI soma of 1d and 4d old animals of the indicated genotypes grown at 20°C (left) and 25°C (right). Both transgenes were expressed under srg-47 promoter sequences. *** indicates different from 1d of the same genotype at P<0.001 (Wilcoxon Mann-Whitney U test). n>45 each; 3 independent experiments. C) Large and small aggregates of SOD1(G85R)::YFP protein in body wall muscle and ASI soma in 1d and 4d old wild-type and osm-6(p811) mutants. Expression was driven under the unc-54 (muscle) and srg-47 promoters (ASI). Large and small SOD1(G85R)::YFP aggregates were defined as puncta that were >3 μm and <1 μm in diameter, respectively. Scale bar: 10 μm. (D) Average number of small and medium sized aggregates of SOD1(G85R)::YFP in the ASI soma of 1d and 4d old animals of the indicated genotypes. Expression in ASI was driven under the srg-47 promoter. Animals also expressed SOD1(G85R)::YFP under muscle specific unc-54 regulatory sequences (see S5C Fig). Small and medium SOD1(G85R)::YFP aggregates were defined as puncta that were <1 μm and between 1–3 μm in diameter, respectively. ## and # indicate different from 1d within a genotype at P<0.005 and 0.05, respectively (Wilcoxon Mann-Whitney U test). n>30 each; 3 independent experiments. E) Quantification of srg-47p::TagRFP levels in ASI neurons of wild-type and osm-6(p811) animals of the indicated ages. n>45 neurons each; 3 independent experiments. AU—arbitrary fluorescence units. (TIF) [file pgen.1006325.s005.tif]

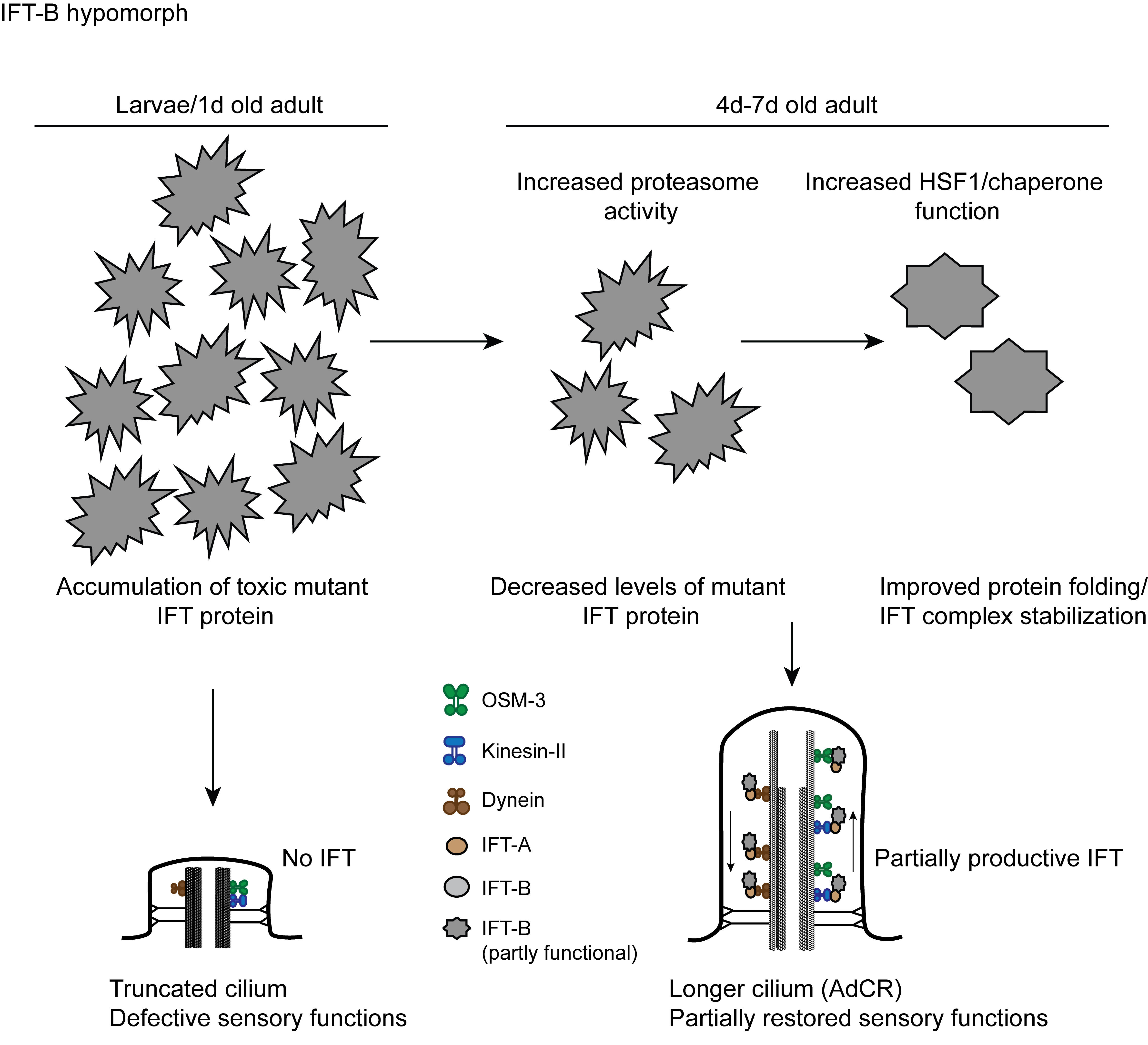

Supplement: S6 Fig — In larvae/young adults, accumulation of a partially functional IFT-B protein disrupts IFT resulting in a truncated sensory cilium and defective chemosensation. In middle-aged adults, degradation of mutant IFT proteins by increased proteasome activity, and improved protein folding or stabilization of the IFT complex by HSF1/chaperones, may lead to partially functional IFT, AdCR, and improved chemosensory behaviors. The function of OSM-3 may be also altered in aged IFT mutant animals. See text for additional details. (TIF) [file pgen.1006325.s006.tif]
