# Supplementary material for: Structural and Functional Recovery of Sensory Cilia in C. elegans IFT Mutants upon Aging
Source: PLoS Genet. 2016 Dec 1;12(12):e1006325. doi: 10.1371/journal.pgen.1006325 (PMC5131903; doi:10.1371/journal.pgen.1006325)
Supplement: S1 Table — (DOCX) [file pgen.1006325.s007.docx]

**S1 Table.** Average velocities of aged animals.

| Genotype | Age (adult) | Average velocity (mm/sec ± SEM) |
| --- | --- | --- |
| Wild-type | 1d  7d | 0.16 ± 0.0  0.08 ± 0.0 |
| *osm-5(p813)* | 1d  7d | 0.13 ± 0.0  0.05 ± 0.0 |
| *osm-6(p811)* | 1d  7d | 0.12 ± 0.0  0.07 ± 0.0 |

Velocities were measured on food-free plates at 20°C.

Animals were grown at 20°C. n = 100 animals each; 5 independent assays.
