# Supplementary material for: Structural and Functional Recovery of Sensory Cilia in C. elegans IFT Mutants upon Aging
Source: PLoS Genet. 2016 Dec 1;12(12):e1006325. doi: 10.1371/journal.pgen.1006325 (PMC5131903; doi:10.1371/journal.pgen.1006325)
Supplement: S2 Table — (DOCX) [file pgen.1006325.s008.docx]

**S2 Table.** Anterograde IFT velocities in ASH/ASI cilia.

| Fusion protein^a^ | Strain^b^ | Adult  age | Mean anterograde velocity (μm/sec ± SD)^c^ | | | |
| --- | --- | --- | --- | --- | --- | --- |
|  |  |  | Middle segment | n/N | Distal segment | n/N |
| KAP-1::GFP | WT | 1d | 0.73 ± 0.10 | 157/8 | - | - |
| KAP-1::GFP | WT | 7d | 0.71 ± 0.12 | 162/8 | - | - |
| KAP-1::GFP | *osm-6* | 7d | 0.70 ± 0.12 | 168/9 | - | - |
| OSM-3::GFP | WT | 1d | 0.69 ± 0.12 | 162/8 | 1.06 ± 0.16 | 150/8 |
| OSM-3::GFP | WT | 7d | 0.75 ± 0.10^d^ | 221/8 | 1.04 ± 0.18 | 180/10 |
| OSM-3::GFP | *osm-6* | 7d | 0.67 ± 0.15^e^ | 159/12 | ND | ND |

^a^Fusion proteins were expressed under the *sra-6* promoter in the ASH/ASI neurons.

^b^The *osm-6(p811)* allele was used.

^c^IFT could not be quantified in the severely truncated cilia of 1d old *osm-6* mutants. Partial elongation of cilia in 7d old *osm-6* animals allowed for IFT analyses only in the middle segments.

^d^indicates different from corresponding 1d old wild-type at *P*<0.05.

^e^indicates different from 7d old wild-type at corresponding age at *P*<0.001.

Analyses were performed in adult animals grown at 20°C. ND: not determined; n: number of GFP particles; N: number of cilia.
