# Supplementary material for: Structural and Functional Recovery of Sensory Cilia in C. elegans IFT Mutants upon Aging
Source: PLoS Genet. 2016 Dec 1;12(12):e1006325. doi: 10.1371/journal.pgen.1006325 (PMC5131903; doi:10.1371/journal.pgen.1006325)
Supplement: S3 Table — (DOCX) [file pgen.1006325.s009.docx]

**S3 Table.** List of strains used in this work.

| Strain | Genotype | Source  and/or  parent strains |
| --- | --- | --- |
| PY3453 | *oyIs50*[*ceh-36*p::*gfp*] | [1] |
| PY6348 | Ex[*sra-6*p::*kap-1::gfp*; *unc-122*p::*dsRed*] | [2] |
| PY6357 | Ex[*sra-6*p::*osm-3::gfp*; *unc-122*p::*dsRed*] | [2] |
| PY8546 | *osm-6(p811)* V; Ex[*str-3*p::*srg-36*::*gfp*; *elt-2*p::*gfp*] | PY8666, PR811 |
| PY8547 | *osm-3(p802)* IV; Ex[*str-3*p::*srg-36*::*gfp*; *elt-2*p::*gfp*] | PY8666, PR802 |
| PY8548 | *daf-10(e1387)* IV; Ex[*str-3*p::*srg-36*::*gfp*; *elt-2*p::*gfp*] | PY8666, [CB1387](http://www.wormbase.org/species/c_elegans/strain/CB1387) |
| PY8549 | *kap-1(ok676)* III; Ex[*str-3*p::*srg-36*::*gfp*; *elt-2*p::*gfp*] | PY8666, RB849 |
| PY8550 | *osm-1(p808)* X; Ex[*str-3*p::*srg-36*::*gfp*; *elt-2*p::*gfp*] | PY8666, PR808 |
| PY8551 | *osm-5(p813)* X; Ex[*str-3*p::*srg-36*::*gfp*; *elt-2*p::*gfp*] | PY8666, PR813 |
| PY8552 | *kap-1(ok676)* III; *osm-3(p802)* IV; Ex[*str-3*p::*srg-36*::*gfp*; *elt-2*p::*gfp*] | PY8547, PY8549 |
| PY8553 | *kap-1(ok676)* III; *osm-6(p811)* V; Ex[*str-3*p::*srg-36*::*gfp*; *elt-2*p::*gfp*] | PY8546, PY8549 |
| PY8554 | *osm-3(p802)* IV; *osm-6(p811)* V; Ex[*str-3*p::*srg-36*::*gfp*; *elt-2*p::*gfp*] | PY8546, PY8547 |
| PY8556 | *osm-6(m533)* V; Ex[*str-3*p::*srg-36* cDNA::*gfp*; *elt-2*p::*gfp*] | PY8666, SP1733 [3] |
| PY8559 | *osm-1(p816)* X; Ex[*str-3*p::*srg-36*::*gfp*; *elt-2*p::*gfp*] | PY8666, PR816 |
| PY8560 | *osm-5(ok451)* X; Ex[*str-3*p::*srg-36*::*gfp*; *elt-2*p::*gfp*] | PY8666, VC265 |
| PY8564 | *osm-6(p811)* V; *oyIs50*[*ceh-36*p::*gfp*] | PY3453, PY8666 |
| PY8571 | *osm-6(p811)* V; Ex[*sra-6*p::*osm-3::gfp*; *unc-122*p::*dsRed*] | PY6357, PY8666 |
| PY8572 | *osm-6(p811)* V; Ex[*sra-6*p::*kap-1::gfp*; *unc-122*p::*dsRed*] | PY6348, PY8666 |
| PY8573 | *daf-16(mu86)* I; Ex[*str-3*p::*srg-36*::*gfp*; *elt-2*p::*gfp*] | PY8666, CF1038 |
| PY8574 | *daf-16(mu86)* I; *osm-6(p811)* V; Ex[*str-3*p::*srg-36*::*gfp*; *elt-2*p::*gfp*] | PY8546, PY8573 |
| PY8575 | *hsf-1(sy441)* I; *osm-6(p811)* V; Ex[*str-3*p::*srg-36*::*gfp*; *elt-2*p::*gfp*] | PY8546, PY8576 |
| PY8576 | *hsf-1(sy441)* I; Ex[*str-3*p::*srg-36*::*gfp*; *elt-2*p::*gfp*] | PY8666, PS3551 |
| PY8579 | *daf-2(e1370)* III; *osm-6(p811)* V; Ex[*str-3*p::*srg-36*::*gfp*; *elt-2*p::*gfp*] | PY8546, PY8580 |
| PY8580 | *daf-2(e1370)* III; Ex[*str-3*p::*srg-36*::*gfp*; *elt-2*p::*gfp*] | PY8666, CB1370 |
| PY8582 | Ex[*srg-47*p::*TagRFP*; *unc-122*p::*dsRed*] | from M. O’Donnell |
| PY8583 | Ex[*sra-6*p::*gfp*; *unc-122*p::*gfp*] | from S. Mukhopadhyay |
| PY8585 | *osm-6(p811)* V; *osm-5(p813)* X; Ex[*str-3*p::*srg-36*::*gfp*; *elt-2*p::*gfp*] | PY8546, PY8548 |
| PY8586 | *daf-10(e1387)* IV; *osm-6(p811)* V; Ex[*str-3*p::*srg-36*::*gfp*; *elt-2*p::*gfp*] | PY8546, PY8551 |
| PY8595 | Ex[*sra-6*p::*gfp*; *unc-122*p::*gfp*]; Ex[*sra-6*p::*mksr-2::TagRFP*; *unc-122*p::*dsRed*] | PY8804, PY8583 |
| PY8596 | *osm-6(p811)* V; Ex[*sra-6*p::*gfp*; *unc-122*p::*gfp*]; Ex[*sra-6*p::*mksr-2::TagRFP*; *unc-122*p::*dsRed*] | PY8595, PR811 |
| PY8597 | Ex[*srg-47*p::*TagRFP*; *unc-122*p::*dsRed*]; Ex[*srg-47*p::*mks-5::gfp*; *unc-122*p::*gfp*] | PY8582, PY8854 |
| PY8598 | *osm-6(p811)* V; Ex[*srg-47*p::*TagRFP*; *unc-122*p::*dsRed*]; Ex[*srg-47*p::*mks-5::gfp*; *unc-122*p::*gfp*] | PY8597, PR811 |
| PY8666 | Ex[*str-3*p::*srg-36* cDNA::*gfp*; *elt-2*p::*gfp*] | [4] |
| PY8804 | Ex[*sra-6*p::*mksr-2::TagRFP*; *unc-122*p::*dsRed*] | from I. Nechipurenko |
| PY8854 | Ex[*srg-47*p::*mks-5::gfp*; *unc-122*p::*gfp*] | from I. Nechipurenko |
| PY9516 | *osm-5(p813)* X; Ex[*sra-6*p::*gfp*; *unc-122*p::*gfp*] | PY8583, PR813 |
| PY9517 | *daf-21(p673)* V; Ex[*sra-6*p::*gfp*; *unc-122*p::*gfp*] | PY8583, JT6130 |
| PY9518 | *daf-21(p673)* V; *osm-5(p813)* X; Ex[*sra-6*p::*gfp*; *unc-122*p::*gfp*] | PY9516, PY9517 |
| PY9519 | *hsf-1(sy441)* I; Ex[*srg-47*p::*TagRFP*; *unc-122*p::*dsRed*] | PY8582, PS3551 |
| PY9520 | *hsf-1(sy441)* I; *osm-6(p811)* V; Ex[*srg-47*p::*TagRFP*; *unc-122*p::*dsRed*] | PY8598, PY9519 |
| PY9526 | Ex[*srg-47*p::*hsf-1::gfp*; *unc-122*p::*gfp*]; Ex[*srg-47*p::*TagRFP*; *unc-122*p::*dsRed*] | PY8582 |
| PY9529  (Line 1) | *hsf-1(sy441)* I; *osm-6(p811)* V; Ex[*srg-47*p::*hsf-1::gfp*; *unc-122*p::*gfp*]; Ex[*srg-47*p::*TagRFP*; *unc-122*p::*dsRed*] | PY9520 |
| PY9534  (Line 2) | *hsf-1(sy441)* I; *osm-6(p811)* V; Ex[*srg-47*p::*hsf-1::gfp*; *unc-122*p::*gfp*]; Ex[*srg-47*p::*TagRFP*; *unc-122*p::*dsRed*] | PY9520 |
| PY10400 | Ex[*str-3*p::*srg-36* cDNA::*gfp*; *elt-2*p::*gfp*]; Ex[*srg-47*p::*daf-21* dsRNA; *unc-122*p::*mCherry*] | PY8666 |
| PY10403  (Line 1) | *osm-6(p811)* V; Ex[*str-3*p::*srg-36* cDNA::*gfp*; *elt-2*p::*gfp*]; Ex[*srg-47*p::*daf-21* dsRNA; *unc-122*p::*mCherry*] | PY8546 |
| PY10404  (Line 2) | *osm-6(p811)* V; Ex[*str-3*p::*srg-36* cDNA::*gfp*; *elt-2*p::*gfp*]; Ex[*srg-47*p::*daf-21* dsRNA; *unc-122*p::*mCherry*] | PY8546 |
| PY10406 | Ex[*str-3*p::*srg-36* cDNA::*gfp*; *elt-2*p::*gfp*]; Ex[*srg-47*p::*hsf-1* dsRNA, *unc-122*p::*mCherry*] | PY8666 |
| PY10407  (Line 1) | *osm-6(p811)* V; Ex[*str-3*p::*srg-36* cDNA::*gfp*; *elt-2*p::*gfp*]; Ex[*srg-47*p::*hsf-1* dsRNA, *unc-122*p::*mCherry*] | PY8546 |
| PY10408  (Line 2) | *osm-6(p811)* V; Ex[*str-3*p::*srg-36* cDNA::*gfp*; *elt-2*p::*gfp*]; Ex[*srg-47*p::*hsf-1* dsRNA, *unc-122*p::*mCherry*] | PY8546 |
| PY10409  (Line 1) | Ex[*srg-47*p::*osm-6(p811)* gDNA::*gfp::SL2::mCherry*; *unc-122*p::*gfp*] |  |
| PY10410  (Line 2) | Ex[*srg-47*p::*osm-6(p811)* gDNA::*gfp::SL2::mCherry*; *unc-122*p::*gfp*] |  |
| PY10411 | Ex[*srg-47*p:*osm-5-GFP::SL2::mCherry* *unc-122*p::*gfp*] |  |
| PY10412 | *osm-5(ok451)* X; Ex[*srg-47*p:*osm-5-gfp::SL2::mCherry*; *unc-122*p::*gfp*] | PY10411, VC265 |
| PY10413 | Ex[*srg-47*p:*osm-5-p813-gfp*; *srg-47*p*::TagRfp*; *unc-122*p::*gfp*] |  |
| PY10414 | *osm-5(p813)* X; Ex[*srg-47*p:*osm-5-p813-gfp*; *srg-47*p*::TagRfp*; *unc-122*p::*gfp*] | PR813, PY10413 |
| PY10415 | *osm-5(p813)* X; Ex[*srg-47*p*::TagRfp*; *unc-122*p::*gfp*] |  |
| PY10416 | Ex[*srg-47*p::*Sod1(G85R)::yfp*; *unc-54*p::*Sod1(G85R)::yfp*] |  |
| PY10417 | *osm-6(p811)* V; Ex[*srg-47*p::*Sod1(G85R)::yfp*; *unc-54*p::*Sod1(G85R)::yfp*] | PR811, PY10416 |
| PY10418 | Ex[*srg-47*p::*Ub-G76::gfp*, *srg-47*p::*TagRfp*; *unc-122*p::*gfp*] |  |
| PY10419 | *osm-6(p811)* V; Ex[*srg-47*p::*Ub-G76V::gfp*, *srg-47*p::*TagRfp*; *unc-122*p::*gfp*] | PR811, PY10418 |
| PY11400 | *osm-5(p813)* X; *oyIs14(sra-6*p*::gfp) V* | PY1058, PR811 |
| PY11401 | *osm-6(p811)* V; Ex[*sra-6*p*::gfp*] | PR811 |
| TJ375 | *gpIs1(hsp-16.2::gfp)* | [5] |
| PY11402 | *osm-5(p813); gpIs1(hsp-16.2::gfp)* | PR813, TJ375 |

**REFERENCES**

1. Kim K, Kim R, Sengupta P. The HMX/NKX homeodomain protein MLS-2 specifies the identity of the AWC sensory neuron type via regulation of the *ceh-36 Otx* gene in *C. elegans*. Development. 2010;137: 963-974.

2. Mukhopadhyay S, Lu Y, Qin H, Lanjuin A, Shaham S, Sengupta P. Distinct IFT mechanisms contribute to the generation of ciliary structural diversity in *C. elegans* EMBO J. 2007;26: 2966-2980.

3. Starich TA, Herman RK, Kari CK, Yeh W-H, Schackwitz WS, Schuyler MW, et al. Mutations affecting the chemosensory neurons of *Caenorhabditis elegans*. Genetics. 1995;139: 171-188.

4. McGrath PT, Xu Y, Ailion M, Garrison JL, Butcher RA, Bargmann CI. Parallel evolution of domesticated *Caenorhabditis* species targets pheromone receptor genes. Nature. 2011;477: 321-325.

5. Rea SL, Wu D, Cypser JR, Vaupel JW, Johnson TE. A stress-sensitive reporter predicts longevity in isogenic populations of *Caenorhabditis elegans*. Nat Genet. 2005;37: 894-898.
